# Technical development of two-photon optogenetic stimulation and its potential application to brain–machine interfaces

**DOI:** 10.1117/1.NPh.13.1.010601

**Published:** 2026-03-04

**Authors:** Riichiro Hira, Yoshikazu Isomura

**Affiliations:** a Institute of Science Tokyo, Graduate School of Medical and Dental Sciences, Department of Physiology and Cell Biology, Tokyo, Japan; b RIKEN, Center for Computational Science, High Performance Artificial Intelligent System Research Team, Saitama, Japan

**Keywords:** two-photon optogenetics, large field of view two-photon calcium imaging, brain machine interface

## Abstract

Over the past decade, techniques enabling bidirectional modulation of neuronal activity with single-cell precision have rapidly advanced in the form of two-photon optogenetic stimulation. Unlike conventional electrophysiological approaches or one-photon optogenetics, which inevitably activate many neurons surrounding the target, two-photon optogenetics can drive hundreds of specifically targeted neurons simultaneously, with stimulation patterns that can be flexibly and rapidly reconfigured. In this review, we trace the development of two-photon optogenetic stimulation, focusing on its progression toward implementations in large field of view two-photon microscopes capable of targeted multi-neuron control. We highlight three principal strategies: spiral scanning, temporal focusing, and three-dimensional computer-generated holography, along with their combinations, which together provide powerful tools for causal interrogation of neural circuits and behavior. Finally, we discuss the integration of these optical technologies into brain–machine interfaces, emphasizing both their transformative potential and the technical challenges that must be addressed to realize their broader impact.

## 1 Introduction

**Fig. 1:**
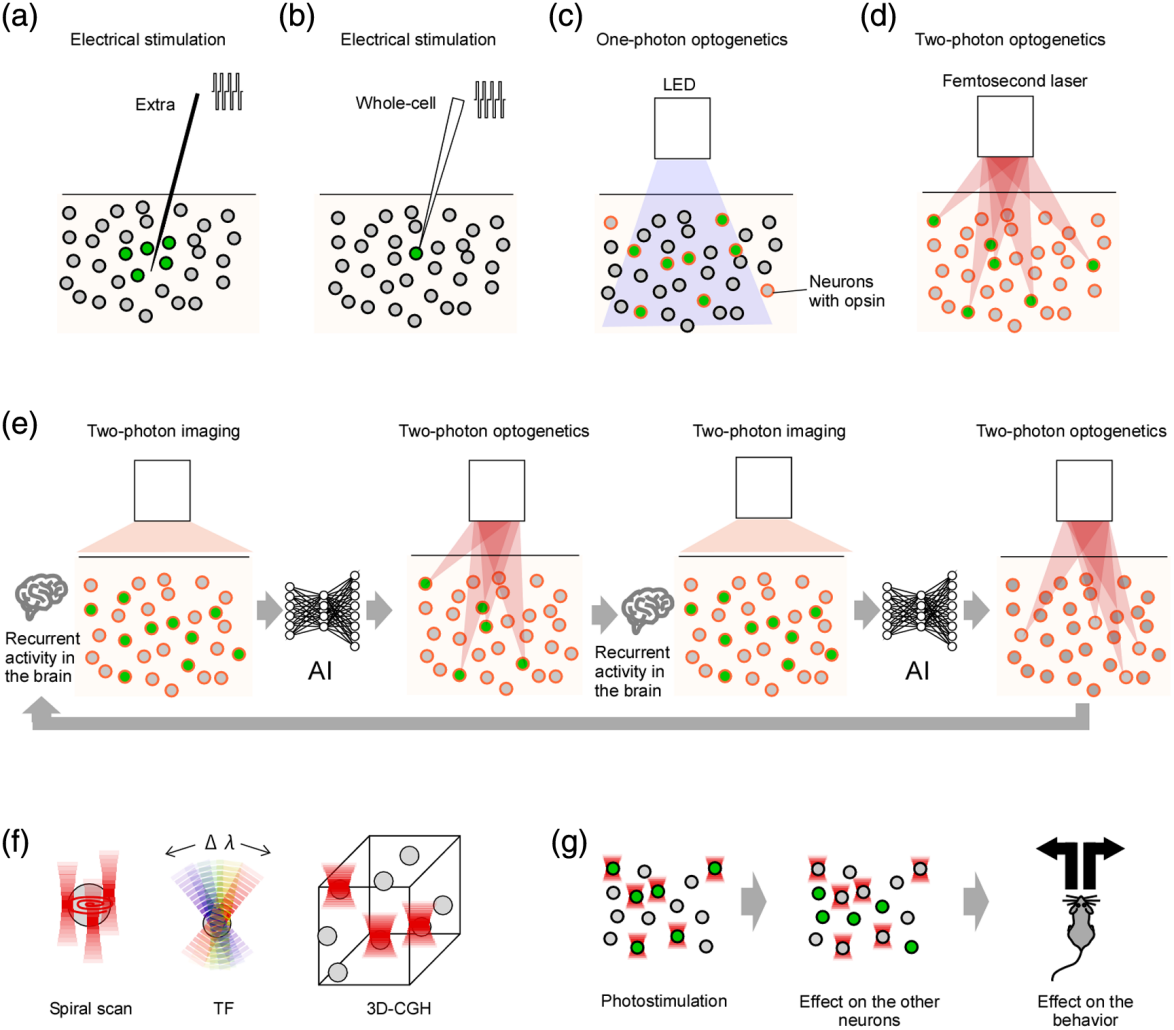
Mechanisms and purposes of two-photon optogenetic stimulation of neurons. (a) Extracellular electrical stimulation with electrodes activates cells near the stimulating site, and if axons run nearby, they may also be recruited. (b) When current is injected through a whole-cell patch clamp, stimulation can be confined to a single neuron, although the number of neurons that can be patched simultaneously is limited to only a few. (c) One-photon optogenetic stimulation, based on opsin expression and light delivery, can spatially restrict activation to opsin-expressing cells, but it cannot be confined to a precisely targeted single cell. (d) Two-photon optogenetic stimulation overcomes this limitation: owing to the nonlinearity of two-photon absorption, opsin expression does not need to be spatially restricted, and arbitrary stimulation patterns can be generated. (e) Two-photon calcium imaging can be used to monitor brain activity and feed it into AI, which in turn guides two-photon optogenetic stimulation. Iterating this closed-loop interaction between brain and AI may open new directions for brain–machine interfaces. (f) Three major strategies for two-photon optogenetic stimulation have been developed—spiral scanning, TF, and 3D-CGH. (g) By selectively stimulating single cells or ensembles and monitoring the effects on non-targeted cells and behavior, researchers can directly probe causal relationships between neural circuits and behavior at the ensemble level.

Traditionally, methods for stimulating neurons in the living brain have relied on electrophysiological techniques. For example, extracellular electrical stimulation using electrodes is still employed to probe the function of specific brain regions, but it nonspecifically activates cells and axons located near the electrode tip [Fig. 1(a)]. Whole-cell patch clamp, by forming a gigaohm seal between the glass electrode and the cell membrane, prevents current leakage and allows stimulation to be strictly confined to a single targeted neuron [Fig. 1(b)]. However, performing whole-cell patch clamp *in vivo* is technically challenging, and it is virtually impossible to apply this method to more than a few neurons simultaneously.

Optogenetic stimulation with one-photon illumination enables finer control by restricting opsin expression to specific cell types and limiting the illumination area [Fig. 1(c)]. Nonetheless, because of the nature of one-photon excitation, stimulation cannot be confined to a single targeted cell, and arbitrary combinations of cells cannot be addressed. Two-photon optogenetic stimulation overcomes this limitation. Due to the nonlinearity of two-photon absorption, it is possible to stimulate targeted ensembles of cells, and this can be achieved for arbitrary combinations [Fig. 1(d)]. Such arbitrary spatiotemporal stimulation patterns cannot be realized by any method other than two-photon optogenetics, which explains why this unique technique has rapidly advanced over the past 15 years.

If arbitrary combinations of cells can be stimulated with two-photon light, what new possibilities emerge for brain–machine interfaces (BMIs)? One can envision a system in which two-photon calcium imaging is used to monitor neural activity in real time, the recorded signals are fed into an artificial intelligence algorithm, and the artificial intelligence (AI) then determines which combination of cells should be stimulated next via two-photon optogenetics [Fig. 1(e)]. The resulting activity is again measured by two-photon calcium imaging, completing a closed-loop interaction. If such closed-loop control could be implemented at scale and speed, BMIs would be transformed into something entirely new—not merely reading information from the brain but enabling the brain and AI to work as a unified system to augment brain function. In this article, we provide a detailed survey of two-photon optogenetic stimulation methods, which are expected to underpin such advanced applications.

### 1.1 Optogenetics and Two-Photon Excitation

Two-photon excitation is essentially absent in the natural environment, so opsins such as ChR2 did not evolve for this purpose. Nevertheless, ChR2 can still be excited by two photons at approximately twice the wavelength (∼470  nm×2) of its single-photon activation band. In experimental settings, femtosecond pulsed lasers in the near-infrared range (900 to 1100 nm) are used to drive opsins through two-photon absorption. Because the probability of two-photon absorption scales with the square of photon density, excitation is confined to a tiny focal volume deep within brain tissue. By appropriately shaping the size and geometry of this focal region, it becomes possible to selectively stimulate specific neuronal populations. Techniques that achieve such focal shaping include spiral scanning (tracing the cell perimeter in a spiral to stimulate the entire membrane evenly), temporal focusing, and computer-generated holography (CGH) [Fig. 1(f)]. The primary purpose of applying these stimulation methods *in vivo* to animal brains is to investigate the causal effects of specific stimulation on distinct cell populations and their impact on behavior [Fig. 1(g)]. As no other method exists to achieve this except for two-photon optogenetics, two-photon optogenetic stimulation remains a challenging yet promising technique for future development.

### 1.2 Differences Between Two-Photon Optogenetic Stimulation and Two-Photon Fluorescence Imaging

Two-photon calcium imaging has become a widely used approach for monitoring neuronal activity during behavior.^1^ The most commonly used fluorophore for this purpose is G-CaMP or GCaMP,^2^^,^^3^ a GFP variant engineered to respond to changes in intracellular ${\mathrm{Ca}}^{2+}$ concentration. GFP and GCaMP emit fluorescence within 5 ns after excitation. This ∼5-ns fluorescence lifetime is shorter than the 12.5-ns pulse interval of the 80-MHz ultrafast lasers typically used for two-photon excitation. As a result, regardless of whether the previous pulse has already excited the fluorophore, the molecule is ready to be re-excited by the next pulse. This property makes 80-MHz pulse trains highly efficient for fluorescence imaging.

By contrast, in two-photon optogenetic stimulation, the excited molecule is an opsin such as ChR2. Upon excitation, the ion channel undergoes a conformational change that opens an ionic pore and then closes again, with opening and closing kinetics on the order of 10 ms. Compared with the ∼5-ns fluorescence lifetime, this corresponds to a difference of about six orders of magnitude. During this 10-ms period, an 80-MHz laser delivers roughly one million pulses. If the ion channel is opened by the first pulse, the subsequent pulses provide no additional effect. Therefore, the laser pulses must be delivered at a much lower repetition rate. This vast disparity in time scales underlies the fundamental distinction in the choice of optimal light sources for imaging versus stimulation.

The differences between two-photon fluorescence imaging and two-photon optogenetic stimulation are not only temporal but also spatial. Imaging biological specimens typically requires submicron, near-diffraction-limited resolution to visualize intracellular structures and the fine morphology of dendritic spines and axons. In contrast, when two-photon optogenetic stimulation is applied *in vivo*, the target is often a single cell. In such cases, a resolution of ∼10  μm, corresponding to the size of a soma, is sufficient. If the stimulation spot is ∼1  μm in diameter, it cannot cover an entire soma and must be scanned across the membrane. Conversely, if the lateral focus is broadened to ∼10  μm by spatial focusing alone, the axial confinement of two-photon excitation is severely compromised. Thus, precise three-dimensional control of the excitation volume is required, creating fundamental optical differences between imaging and stimulation.

**Fig. 2:**
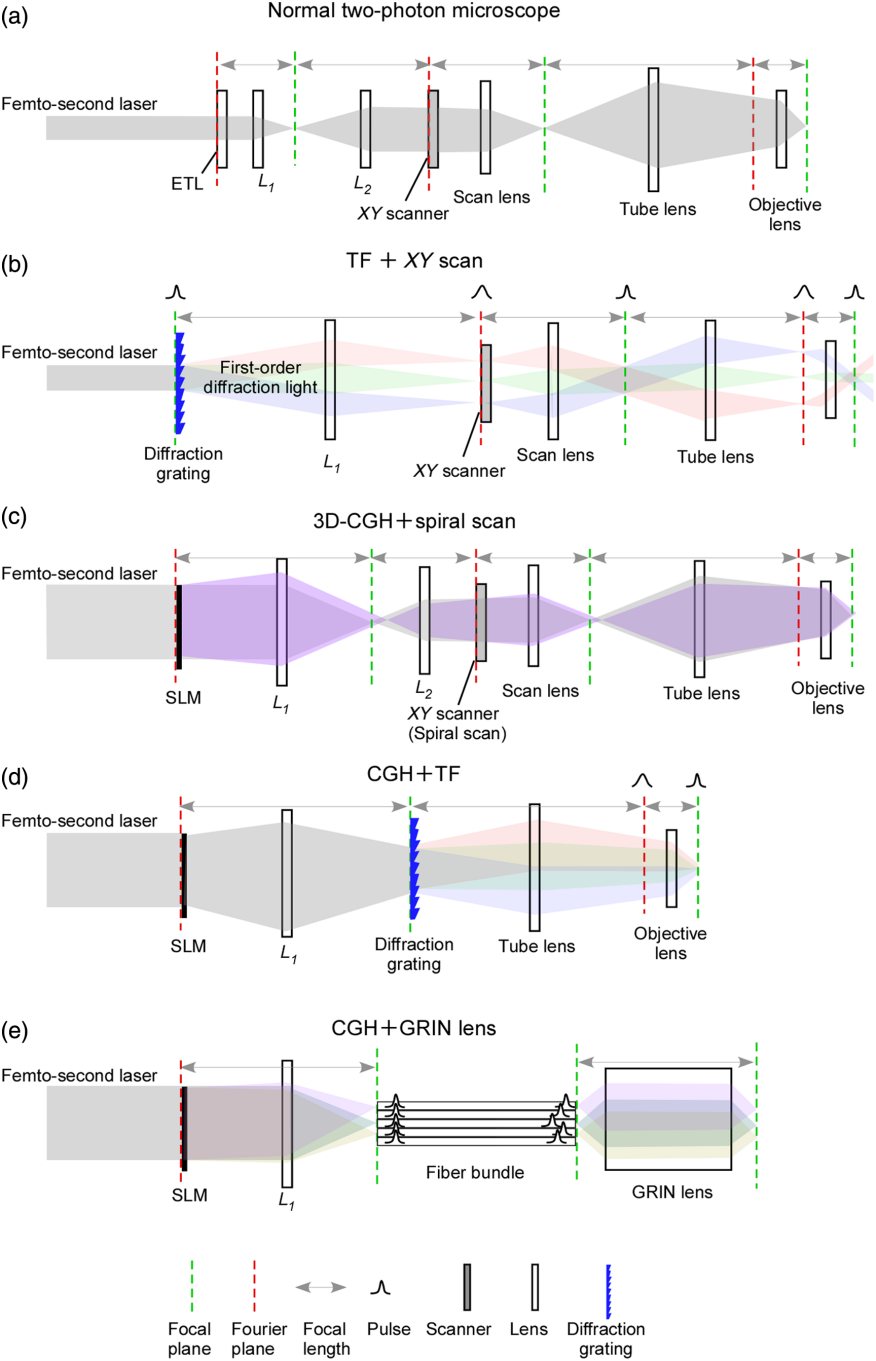
Methods of two-photon optogenetics. (a) Standard two-photon microscope primarily used for imaging. A variable-focus lens at the pupil (Fourier) plane controls the axial position, whereas XY scanners (galvo or resonant) move the lateral focus. Although this architecture can in principle be used for three-dimensional two-photon optogenetic stimulation, ChR2 activation typically saturates under standard conditions. (b) Two-photon stimulation by temporal focusing. A grating conjugate to the focal plane disperses wavelengths at different angles, which recombine only at the focal plane to restore a short pulse. As a result, two-photon excitation occurs only near the focal plane, thereby improving axial resolution. (c) Two-photon stimulation using three-dimensional holography. An SLM placed at the pupil (Fourier) plane generates stimulation spots on the focal plane (gray) and, by adding spherical phase, at different depths (purple). Spiral scanning with galvo mirrors expands each holographic spot to the size of a soma, efficiently activating opsins across the membrane. (d) Two-photon stimulation combining holography and temporal focusing. To stimulate without scanners, the holographic focal volume itself must approximate the size of a soma. However, if the lateral FWHM is extended to ∼10  μm by spatial focusing alone, axial resolution collapses. By placing a grating conjugate to the focal plane after the SLM, spectral dispersion ensures that temporal focusing occurs only at the focal plane, thereby restoring axial resolution. However, only the plane conjugate to the grating undergoes temporal focusing, limiting stimulation to two dimensions per exposure. (e) Transferring a 2D hologram through a fiber bundle and GRIN lens to the brain, ∼2  m bundles exhibit picosecond-scale inter-fiber delay variations, suppressing interference and improving two-photon specificity.

Figure 2(a) schematically shows the optical path of a typical two-photon microscope for imaging. Images are obtained by scanning the focus in three-dimensional (3D): an electro-tunable lens placed at a pupil (Fourier) plane controls axial position and XY scanners (galvo or resonant) control lateral scanning. With this configuration, three-dimensional two-photon optogenetic stimulation is also possible, but per-cell dwell times of ≥10  ms, together with additional focus travel times of ∼10  ms among cells, are often too slow. Although this may be sufficient for certain experiments, it is clearly inadequate when the goal is to activate dozens of assemblies simultaneously or to investigate spike timing-dependent plasticity, where millisecond-scale temporal precision is essential.^4^

## 2 Development of Two-Photon Optogenetic Stimulation

This section outlines several approaches to two-photon optogenetic stimulation. Figure 1(f) illustrates three fundamental techniques: spiral scanning, temporal focusing (TF), and three-dimensional computer-generated holography (3D-CGH). Many studies employ one of these methods or combinations thereof. In doing so, they probe causal relationships between neural circuits and behavior by examining how stimulation affects non-targeted cells and behavioral outputs [Fig. 1(g)].

In the following, we describe the principles and strategies underlying each technique (and their combinations), followed by brief summaries of representative methods and findings from individual studies. The presentation is arranged roughly chronologically, beginning with early demonstrations and progressing toward increasingly sophisticated combinations designed to meet the distinct spatiotemporal requirements of two-photon excitation for imaging versus stimulation. The issue of crosstalk between two-photon optogenetic stimulation and two-photon calcium imaging is also addressed at the end of this section. This section partially overlaps with several prior excellent reviews, and we encourage readers to refer to those works as well.^5^

### 2.1 Spiral Scanning

Six years after the first report of one-photon optogenetic stimulation,^6^ the first study on two-photon optogenetic stimulation was published in 2009.^7^ In cultured neurons, they demonstrated that ChR2 could be driven by two-photon excitation to elicit action potentials. The stimulation employed a Ti:sapphire laser at ∼920  nm and 80 MHz, parameters typical for two-photon imaging at the time. As emphasized in the title of their paper, ChR2 activation rapidly saturated within the focal region, whereas fluorophores have fluorescence lifetimes of ≤10  ns, and ChR2 channel kinetics are on the order of ∼10  ms—a difference of approximately six orders of magnitude. Consequently, immediately after two-photon stimulation, most ChR2 molecules within the focal spot are in the open state, and further illumination there becomes effectively meaningless. At the same time, even regions adjacent to the focal volume with slightly lower photon density can still activate ChR2: when the per-pulse opening probability is small but nonzero, the enormous number of pulses eventually induces channel opening. Thus, the spatial resolution of two-photon optogenetic stimulation is constrained by mechanisms distinct from those governing fluorophore excitation. To optimize both specificity and speed, Rickgauer and Tank^7^ reduced the effective numerical aperture (NA) of the objective to axially elongate the PSF, which was then spiral scanned along the cell outline [Fig. 1(f)]. This strategy successfully induced single-cell action potentials.

This pioneering study appears to have set two directions for subsequent work on two-photon opsin excitation: (i) tailoring the spatial region where photon density is sufficient for two-photon activation to match cellular dimensions and (ii) exploring the use of low-repetition-rate lasers to mitigate saturation. The first principle has remained central as CGH approaches have been developed for two-photon microscopes, whereas the second contributed to the trend toward ∼1-MHz high-power lasers.

Among later reports employing spiral scanning to achieve single-cell-resolution stimulation,^8^^,^^9^ one notable *in vivo* application was reported by Chettih and Harvey^10^ for mapping cortical circuits. They repeatedly applied spiral scanning to layer 2/3 neurons in mouse visual cortex (V1) using C1V1^11^ or ChrimsonR,^12^ restricting opsin expression to perisomatic membranes with a KV2.1 motif.^13^ Stimulation was delivered through a Nikon 16×/0.8 NA objective with a 1070-nm Fidelity 2 laser (a ∼2-W, 80-MHz, 100-fs fiber source), whereas imaging was performed at 920 nm. Cells were stimulated at ∼1  cell/s using 12- to 15-μm spiral scans lasting 32 ms, with an average power of ∼52.7  mW; the scan diameter, slightly larger than the soma, accommodated brain motion. Opsin expression was kept sparse, and off-target effects on neighboring neurons were carefully assessed. Stimulation was paired with visual inputs. The authors estimated that ∼2500 trials per pair would be required to detect reliable causal effects. Although detailed results fall outside the scope of this technological overview, one noteworthy finding was that the net effects of optical stimulation were predominantly inhibitory—a theme that has since been echoed in many subsequent experiments.

### 2.2 Temporal Focusing

TF improves the axial resolution of two-photon excitation by placing a diffraction grating or diffuser conjugate to the focal plane.^14^ This configuration stretches pulses away from the focal plane and recompresses them only at the focal plane.^15^^,^^16^ With a diffraction grating, light is spectrally dispersed at planes above and below the focus and recombines only at the focal plane [Fig. 1(f)]. Although gratings are commonly used, diffusers that impose random phase shifts can similarly elongate pulses away from the focal plane, and the two approaches can be combined.^16^^,^^17^

For neuronal applications, the ideal case is when the focal volume in which peak laser power reaches the two-photon activation threshold matches the size of a single neuron. In that situation, membrane-expressed opsins can be activated without scanning, allowing near-instantaneous activation or inhibition. By expanding the beam laterally at the grating, one can create a disc of ∼10 to 20  μm diameter at the objective’s focal plane. If ∼10-μm lateral excitation was attempted using spatial focusing alone, axial resolution would be severely degraded. TF mitigates this problem, enabling soma-sized excitation spots.

Rickgauer et al.^18^ efficiently implemented two-photon optogenetic stimulation with TF as an alternative to spiral scanning [Fig. 2(b)], successfully activating place cell ensembles in hippocampal CA1. The excitation spots were 10 to 15  μm in diameter with an axial full width at half maximum (FWHM) of ∼6  μm. The stimulation laser was a 1064-nm, 5-W source operating at 80 MHz with ∼200-fs pulses, whereas imaging used 920-nm excitation with GCaMP3. Because CA1 has a laminar organization with densely packed neurons in a quasi-two-dimensional sheet, and principal cells lack strong recurrent connectivity, the interpretation of stimulation effects is in some respects more straightforward than in highly recurrent three-dimensional neocortical circuits.

### 2.3 Holography + Spiral Scanning

A liquid crystal on silicon spatial light modulator modulates the phase of the laser wavefront in a position-dependent manner by adjusting the local orientation of liquid crystal molecules to impose phase delays. Typical refresh rates are on the order of tens of milliseconds, whereas the fastest devices can reach millisecond scales (e.g., Meadowlark products). When placed conjugate to the objective pupil (Fourier plane), the spatial light modulator (SLM) generates holograms at the focal plane.^19^ For ensemble stimulation, the goal is to achieve simultaneous two-photon excitation of specific groups of neurons using holographic patterns. The ability to stimulate multiple neurons at once is a unique advantage of the SLM approach. Methods based on spiral scanning or TF alone cannot accomplish this. Moreover, three-dimensional holograms can be readily obtained by adding spherical phase to a two-dimensional pattern (3D-CGH). If one can design patterns that provide sufficient power for two-photon activation of target neurons without affecting others, and switch them rapidly, it becomes possible to test how different ensembles influence local circuit activity and behavior. The strategy introduced here is to generate a holographic spot at each target neuron’s location via the SLM, and then spiral scan each spot to match soma size, thereby achieving effective two-photon optogenetic stimulation [Fig. 2(c)]. This remains the most widely used method today, with numerous applications described below. Over time, the adoption of lower-repetition-rate lasers and techniques for peri-somatic opsin localization has become standard.

As a representative *in vivo* application, Packer et al.^20^ targeted layer 2/3 pyramidal cells in the mouse primary somatosensory (barrel) cortex expressing C1V1. The SLM (7.68  mm×7.68  mm active area, 512×512  pixels; optimized for 1064 nm; Meadowlark/Boulder Nonlinear Systems, Frederick, Colorado, United States) was placed conjugate to the pupil. Stimulation was delivered at 1064 nm (5 W, 350 to 400 fs; Fianium Ltd., Southampton, United Kingdom) or 1055 nm (2.3 W, 100 fs; Coherent, Saxonburg, Pennsylvania, United States). A Gerchberg–Saxton (GS) algorithm^21^ was used to optimize the phase pattern, with pre-compensation to increase peripheral brightness relative to the center, a common practice. Imaging employed GCaMP6s with 920-nm excitation, using a Nikon 16×/0.8 NA water-immersion objective. Spiral scanning (20  μm diameter, 20 ms) enabled simultaneous stimulation of ∼10  cells, with a mean spike latency of ∼17  ms.

Yang et al.^22^ performed three-dimensional multi-neuron stimulation in layer 2/3 of mouse V1 using the opsin C1V1. Stimulation was delivered with a Spirit 1040-8 laser (1040 nm, 400 fs, 40  μJ, 0.2 to 1 MHz; Spectra Physics, Milpitas, California, United States), whereas imaging was conducted at 940 nm. They employed an HSP512-1064 SLM ($7.68\times 7.68\;{\mathrm{mm}}^{2}$ active area, 512×512  pixels; Meadowlark) in combination with an Olympus 25×/1.05 NA XLPlan water-immersion objective. Approximately 50 cells distributed across ∼150  μm depth were targeted, and 78% of them were activated with ∼300  mW for 95 ms, although non-targeted cells within ∼20  μm also showed notable activation. In addition, they expressed C1V1 in SOM interneurons via Cre drivers and selectively stimulated these cells.

Carrillo-Reid et al.^23^ used two-photon holography in mouse V1 to selectively activate learned functional ensembles and modulate visual behavior. They expressed the opsin C1V1 in layer 2/3 pyramidal neurons, and stimulation was delivered at 1040 nm with a 1-MHz laser. Imaging was performed at 940 nm with GCaMP6s using the same Olympus 25×/1.05 NA objective. Stimulation patterns were generated by combining an SLM with spiral scanning, using 12-μm-diameter spirals at 20 Hz with ∼5  mW per cell. During the “go” window of the behavioral task, illumination lasted 2 s. Remarkably, activating only two neurons from a learned ensemble was sufficient to recruit the rest of the ensemble and enhance visual discrimination performance, whereas perturbing the ensemble by stimulating unrelated neurons degraded performance.

In another study, Marshel et al.^24^ employed two-photon holography to reproduce visual percepts by stimulating excitatory ensembles in mouse V1. They introduced ChRmine, an opsin generating exceptionally high photocurrent. Stimulation was delivered with a Monaco 1035-80-60 laser (1035 nm, 10 MHz, 80 W, 300 fs). Imaging was performed at 940 nm. A custom MacroSLM (1536×1536  pixels, 30.7  mm×30.7  mm active area, 20-μm pitch) was used in conjunction with spiral scanning, in which 25-μm-diameter spirals were applied for 4 ms and repeated 12 times. By replaying spatial patterns of naturally evoked activity, they were able to modulate the animals’ behavioral accuracy, with simultaneous targets ranging from several dozen neurons up to ∼100  cells.

Dalgleish et al.^25^ expressed C1V1-KV2.1 in layer 2/3 pyramidal cells of the S1 barrel cortex and applied two-photon stimulation using an SLM combined with spiral scanning. Stimulation was delivered with an Amplitude Satsuma laser operating at 1030 nm, 2 MHz, and 20 W. The SLM was a Meadowlark device with 512×512  pixels and a 7.68  mm×7.68  mm active area. Spiral scans were 10  μm in diameter, with ∼6  mW per cell, and each trial involved ∼5  ms of stimulation. The resulting spatial resolution was ∼5  μm laterally and ∼20  μm axially. By varying the number of neurons stimulated per trial, the authors estimated that synchronous activation of ∼14 neurons was sufficient to overcome network inhibition and improve detection performance.

Fişek et al.^26^ investigated feedback from the higher visual area LM to V1 by stimulating neurons in LM. They expressed C1V1-KV2.1 and used an Amplitude Satsuma HP2 laser operating at 1 MHz, 30 W, 40  μJ, and 400 fs. Two microscope configurations were employed: one with a Nikon 16×/0.8 NA objective and an SLM with 512×512  pixels and a 7.68  mm×7.68  mm active area (BNS/Meadowlark) and another with a Thorlabs 10×/0.5 NA objective and a larger SLM with 1920×1152  pixels and a 17.6  mm×10.7  mm active area (Meadowlark). These provided fields of view of 1215 and 1920  μm, respectively. Stimulation was applied with SLM-guided spiral scanning, delivering ∼12  mW per cell with 16-μm-diameter spirals, using 10-ms pulses at 20 Hz repeated 10 times for a total duration of 500 ms. Functionally, they found that paired LM and V1 neurons with overlapping receptive fields exhibited suppression in V1, whereas pairs with slightly different receptive fields showed excitation, attributable to apical dendritic ${\mathrm{Ca}}^{2+}$ spikes. The top-down suppression observed in the case of shared receptive fields is consistent with predictive coding, whereas the excitation seen with non-identical receptive fields may support context-dependent cooperation. From a technical perspective, their design leveraged ∼1-mm-scale field of view (FOV) to probe inter-areal feedback by combining two-photon stimulation with dendritic imaging.

Russell et al.^27^ assessed the sensitivity of two-photon stimulation in mouse V1 during a contrast detection task. They expressed C1V1-KV2.1 and used a Satsuma laser with a 512×512 SLM, following the setup described in Fişek et al., but explicitly implemented the Bruker NeuraLight 3D system. A three-dimensional holographic pattern combined with spiral scanning delivered 10-μm-diameter, 20-ms stimulations once every 50 ms during the behavioral task. When visual contrast was low and the pupils were dilated, indicating engagement, optical stimulation improved detection. The authors concluded that cortical responses to stimulation depend strongly on behavioral and affective state as well as sensory context.

Wu et al.^28^ targeted the motor cortex using peri-somatically localized ChRmine-KV2.1 delivered with a commercial Bruker two-photon system. Stimulation was performed with a Monaco 1035-40-40 laser (1 MHz, 1035 nm, 40 W, 350 fs), and holograms were generated with a 512×512 SLM operating at an update rate of ∼600  Hz. Spiral scanning was applied with 10  μm-diameter rotations at ∼7  mW per cell. Using the NeuraLight 3D system—also employed in Piantadosi et al., Russell et al., and Vinograd et al.^27^^,^^29^^,^^30^—they demonstrated that stimulating ∼20 early task-related M1 neurons was sufficient to initiate movement, whereas stimulating ∼10 early neurons or late-phase neurons was not. Movement initiation depended on the preparatory state, and stimulation of early neurons recruited additional non-stimulated, movement-related neurons, consistent with a pattern-completion mechanism.

### 2.4 Holography + Temporal Focusing

In earlier approaches, TF and holography were applied separately, with a galvo mirror placed downstream of either the grating or the SLM to direct patterned light to the desired location. Here, we discuss a key advance: directly setting the lateral size of the hologram to match the soma scale, while using temporal focusing to restore axial confinement. This approach enables stimulation without spiral scanning [Fig. 2(d)].

Papagiakoumou et al.^31^^–^^33^ first implemented this strategy by sending light to an SLM to generate a phase pattern, projecting that pattern onto a grating and then achieving temporal focusing at the objective focal plane. Because the SLM and the grating are positioned at Fourier-related planes, simply placing the grating first would cause spectrally separated spots to appear on the SLM, making it apparently impossible to construct a general pattern through wavelength-dependent phase. For this reason, the sequence SLM followed by grating is required.

Chen et al.^34^ demonstrated sub-millisecond jitter and tens-of-hertz spiking in layer 2/3 of V1 using a combination of temporal focusing and holography with ReaChR, CoChR, and ChrimsonR. Stimulation was provided by an Amplitude Satsuma HP laser (1030 nm, 250 fs, 20  μJ; 10 W, 500 kHz), and the SLM was a Hamamatsu X10468-07 (792×600  pixels, 20  μm pitch, rise time 10 ms, fall time 80 ms).

A limitation of the SLM-to-grating configuration is that two-photon excitation is effectively restricted to the two-dimensional pattern formed on the grating plane. Even if the SLM phase pattern is designed as a three-dimensional hologram, spectral dispersion occurs only at the grating-conjugate plane, and temporal focusing is confined to that plane. One possible approach to realizing a three-dimensional hologram is to add spherical phase terms to each two-dimensional layer and sum them. However, outside the grating-conjugate plane, differences in optical path length across wavelengths prevent pulse recompression, making temporal focusing ineffective. As a result, spatial resolution for stimulation at planes other than the conjugate plane deteriorates substantially. The next section describes three-dimensional scanless holographic optogenetics with temporal focusing (3D-SHOT), which overcomes this limitation.

### 2.5 Three-Dimensional Scanless Holographic Optogenetics with Temporal Focusing

As noted, 3D-SHOT combines temporal focusing with SLM-based three-dimensional holography to achieve scanless stimulation of ensembles distributed in three dimensions. In standard holography, all wavelengths follow the same optical path. In contrast, 3D-SHOT introduces a grating that first disperses the spectrum, allowing the SLM to impose wavelength-dependent phase shifts and thereby enabling temporal focusing at each target spot. This approach was proposed and demonstrated in two studies from the Adesnik Lab.^17^^,^^35^ The 2018 implementation that introduced a rotating diffuser (“3D-SHOT 2.0”) is now widely employed, whereas the 2017 study laid out the conceptual framework.

**Fig. 3:**
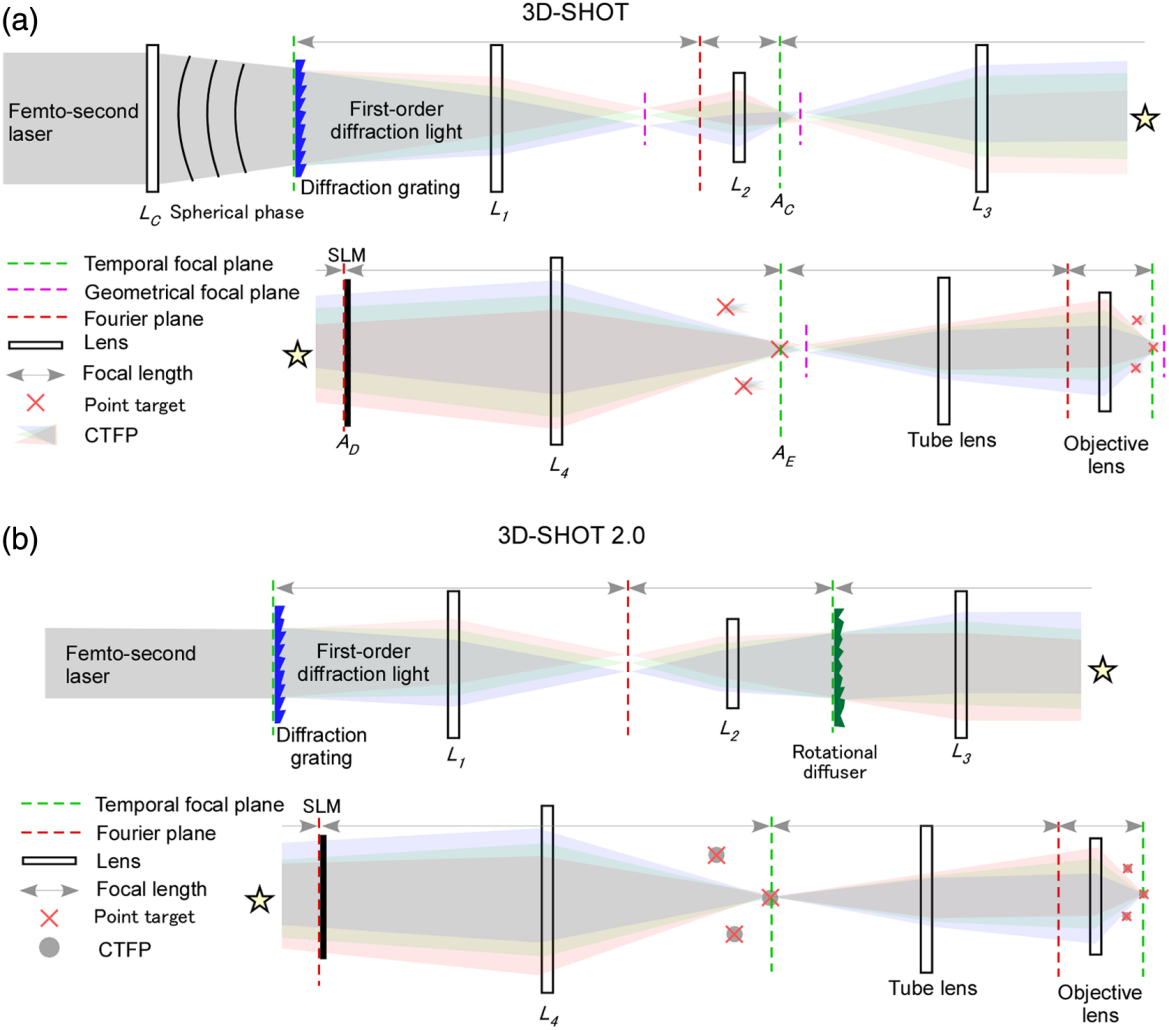
Mechanism of 3D-SHOT. (a) 3D-SHOT optical path. A grating is placed before the SLM. A lens Lc adds spherical phase to the light incident on the grating. By intentionally separating the temporal-focus plane and the geometric focus plane, 3D-CGH is reconciled with TF. The CTFP formed by the grating + Lc is copied by the SLM to arbitrary 3D positions. Because different wavelengths illuminate different SLM pixels, the SLM can adjust phase per wavelength, equalizing path length and realizing TF at each 3D target. (b) 3D-SHOT 2.0. A rotating diffuser substitutes for Lc. The diffuser slightly spreads each wavelength at the SLM, enabling both spatial CGH and per-wavelength phase control to equalize path lengths for TF, whereas speckle is temporally randomized.

Pégard et al.^35^ introduced the theoretical framework and demonstrated the fusion of three-dimensional holography with temporal focusing [Fig. 3(a)]. They used ChrimsonR and Chronos as opsins, restricting their expression to peri-somatic membranes with a KV2.1 motif. A modified multilevel Gerchberg–Saxton algorithm was developed, incorporating analytic expressions for the target phase.

In 3D-SHOT, the grating, together with the preceding lens (Lc), first generates a disk-shaped temporal focusing pattern, referred to as the custom temporal focus pattern (CTFP). The SLM then replicates this CTFP at arbitrary three-dimensional coordinates as a point-cloud hologram. Importantly, the SLM does not sculpt the focal spot itself; it merely places copies of the CTFP at designated 3D positions. The incident light is converted into a spherical wave by Lc before reaching the diffraction grating. The plane of the grating corresponds to the temporal focus plane (green). After the grating, the light is relayed by a 4f system consisting of L1 and L2. The Fourier plane (red) and the temporal focus plane lie between L1 and L2, shifted closer to L2 relative to the geometrical focal plane. This Fourier plane is conjugate to the SLM. The temporal focus plane, labeled Ac, is offset from the subsequent geometric focal plane—an intentional displacement engineered by Lc—which generates a conical region of high photon density (the CTFP). Note that the photon density may split into two peaks if the distance between these planes is large. The SLM is placed, via L3, at the Fourier plane of the temporal focus plane, where a phase pattern is applied. Here, the phase pattern is computed to replicate the CTFP in three dimensions. The SLM thus produces a point-cloud hologram, but unlike conventional holography, different wavelengths strike different SLM pixels, allowing the imposition of wavelength-specific phases. This enables not only ordinary 3D spatial focusing according to the holographic design but also temporal focusing at each target point by equalizing the optical path length across wavelengths. This feature distinguishes 3D-SHOT from simple 3D-CGH. Although the calculations are intricate, details are provided in the supplementary information of the paper. After L4, the replicated CTFPs distributed in 3D are transferred through the tube lens and objective to the neural population, where each site undergoes temporal focusing. The key innovation of 3D-SHOT lies in the arrangement of the grating and Lc, which allows the SLM to be used for combined spatiotemporal focusing.

The next step is an improved version, often referred to as 3D-SHOT 2.0 [Fig. 3(b)]. Mardinly et al.^17^ successfully achieved efficient, bidirectional control of three-dimensionally distributed neuronal ensembles in the mouse cortex. The opsins used were ChroME (an excitatory opsin) or eGtACR1 (an inhibitory, ${\mathrm{Cl}}^{-}$-conducting opsin), each fused to a Kv2.1 trafficking sequence. The excitation sources were either a Coherent Monaco laser (1040 nm, 2 MHz, 40 W) or an Amplitude Satsuma laser (1040 nm, 2 MHz, 20 W). The SLM was a Meadowlark 512 L. A rotating diffuser (θ=0.5  deg, #47-989, Edmund Optics, Barrington, New Jersey, United States) was mounted on a motor with a DVD spindle and served as a replacement for lens Lc.

In 3D-SHOT 2.0, lens Lc is omitted, and the incident beam is directed straight onto the diffraction grating, which is conjugate to the focal plane. After lens L1, the spectrally dispersed light converges at a plane conjugate to the Fourier plane. Downstream of L2, a rotating diffuser is positioned at a plane conjugate to the grating. Here, the diffuser introduces random phase shifts, which mitigate frequency-dependent focusing when projected onto the SLM, placed at the Fourier plane after L3. This reproduces the functionality originally provided by Lc in the first 3D-SHOT implementation. Without the diffuser, the dispersed spectrum would be sharply aligned across the SLM, as at a pupil plane between L1 and L2. With the diffuser, however, light of different frequencies spreads laterally and overlaps on the SLM, as shown in the schematic. Rotation of the diffuser temporally randomizes speckle, reducing unwanted stimulation outside the designed pattern. In addition, because the diffuser broadens the local pulse duration, it contributes to temporal focusing. Thus, without sacrificing temporal focusing by the grating, 3D-SHOT 2.0 introduces a slight spectral spread that allows wavelength-specific phase control at the SLM while retaining ordinary spatial holographic control.

Pattern generation was implemented using either the GS algorithm or the NOVO-CGH algorithm.^36^ With sub-millisecond temporal precision (jitter <1  ms), they were able to simultaneously stimulate up to 50 neurons in 3D, synthesizing arbitrary firing patterns within a volume of 550×550×100  μm.

Below, we introduce three studies that have employed 3D-SHOT. In the study by Sridharan et al.,^37^ the opsin ChroME was engineered to generate several new variants, including ChroME2s. Although the photocurrents of ChroME2s are weaker than those of ChRmine, the faster on/off kinetics enable reliable high-frequency stimulation. Using 3D-SHOT, they succeeded in simultaneously stimulating a total of 631 cells per second in the mouse visual cortex, divided into multiple groups. The excitation sources were a Satsuma HP2 (1030 nm, 2 MHz, 350 fs, Amplitude, San Francisco, California, United States) or a Monaco 1035-80-60 (1040 nm, 1 MHz, 300 fs, Coherent), providing up to 60 W of power. A blazed diffraction grating (600  L/mm, 1000 nm blaze; Edmund Optics 49-570 or Newport 33010FL01-520R) was employed, together with an HSP1920 SLM (192×1152  pixels; Meadowlark Optics). They adopted the 3D-SHOT 2.0 configuration with a rotating diffuser, and the SLM phase patterns were computed using the GS algorithm. ChroME2s exhibited large instantaneous photocurrents and fast response kinetics, making it particularly effective for high-temporal-precision applications. However, in terms of overall sensitivity to two-photon optogenetic stimulation, ChRmine still outperformed ChroME2s.

Oldenburg et al.^38^ used 3D-SHOT to precisely manipulate excitatory populations in layer 2/3 of mouse V1, thereby dissecting recurrent circuit motifs. The opsins were ChroME or ChroME2s. The laser source was a Monaco 40 (1040 nm, 2 MHz), paired with a blazed diffraction grating (Newport R5000626767-19311) and an HSP1920-1064-HSP8-HB SLM (1920×1152  pixels; Meadowlark Optics). The implementation followed the rotating diffuser approach described by Mardinly et al.^17^ Per-cell powers ranged from 12.5 to 100 mW. The achieved axial resolution was ∼10- to 15-μm FWHM, remaining nearly invariant across ∼80  μm of depth. Functionally, ensembles with similar visual tuning but extended spatial distributions tended to recruit additional nearby activity, whereas compact ensembles induced strong local inhibition, demonstrating an interplay between physical arrangement and feature preference.

Bounds and Adesnik^39^ examined how 3D-SHOT-mediated stimulation of V1 ensembles during a visual detection task modulates both surrounding activity and behavior. The opsin was ChroME2s without soma restriction. The optical setup included a Monaco 1035-80-40 laser (1035 nm, 2 MHz, 276 fs), an HSP1920-1064-HSP8-HB SLM (1920×1152  pixels; Meadowlark Optics), and a blazed diffraction grating (Newport 33010FL01-520R). The effective resolution was ∼9.7  μm laterally and ∼20  μm axially (FWHM). They simultaneously stimulated 7 to 40 neurons. Strikingly, targeting the most visually responsive cells alone did not yield a special behavioral advantage. Instead, the behavioral outcome depended on how much additional local activity the stimulation recruited, underscoring the role of ensemble context within cortical networks.

### 2.6 3D-SHOT Combined with Large FOV Two-Photon Microscopy

3D-SHOT is considered one of the definitive methods for fast and flexible three-dimensional stimulation of neuronal ensembles. However, one of the principal limitations on the number of neurons that can be stimulated simultaneously arises from the FOV of the objective lens. A natural way to overcome this constraint is to employ large FOV two-photon microscopes. Several such systems have been developed, including Diesel2p,^40^^–^^42^ 2p-RAM,^43^ and Fashio-2PM.^44^ Here, we introduce a study that applied 3D-SHOT to the 2p-RAM platform.

Abdeladim et al.^5^ integrated the 3D-SHOT optical path into a commercial large FOV two-photon microscope (2p-RAM), which provides an imaging FOV exceeding 5 mm. This implementation enabled three-dimensional holographic photostimulation within an ∼1-mm subregion of the field. The opsins were *ChroME* or *ChroME2s*. The experimental animals included Vglut1-Cre × Ai203 double-transgenic mice,^45^ as well as triple-transgenic mice (EMX1-Cre, CaMK2-tTA, and tetO-GCaMP6s) into which Cre-dependent *ChroME2s* was delivered via AAV. Two-photon stimulation was performed using a fiber laser (Aeropulse 50, NKT Photonics; 1030 nm, ∼1  MHz, 50 W, <500  fs), and GCaMP7s imaging was carried out at 920 nm. A 1920×1152 SLM (Meadowlark Optics) was used. Because the 2p-RAM system employs a vertically mounted rotating breadboard, both stimulation and imaging beams were dynamically aligned via a custom “gantry periscope.” The optical path followed the standard 3D-SHOT configuration: diffraction grating → rotating diffuser → SLM → objective.

The achieved spatial resolution was ∼32  μm laterally and ∼9  μm axially (FWHM), slightly lower than in other 3D-SHOT implementations (20  μm in Mardinly et al.;^17^ 28  μm in Pégard et al.^35^), likely due to the relatively low NA (0.6) of the objective. The effective stimulation range was ∼1  mm×1  mm, which represents only a fraction of the total 2p-RAM FOV. In their experiments, they imaged a ∼3-mm-wide region encompassing primary and multiple higher visual areas while stimulating ensembles in the higher visual area LM. Although some distant neurons showed increased activity, the net effect was predominantly suppressive both within LM and in distal regions. This represents the first study to causally examine inter-areal propagation of activity at the level of neuronal ensembles.

As the work remains at the preprint stage, several technical limitations are notable. The full FOV of 2p-RAM was not exploited, and the stimulation region covered only a subset of the available imaging field. In principle, even with a limited SLM aperture, galvanometric scanning could allow the stimulation field to be repositioned anywhere within the imaging FOV, but this has not yet been achieved. Moreover, 2p-RAM suffers from substantial field curvature, with focal shifts of ∼150  μm at the edges of the imaging field. This may pose a practical challenge for repositioning the stimulation plane across large distances without physically moving the animal.

## 3 Summary and Comparison of Two-Photon Optogenetic Stimulation Methods

In Sec. 2, we introduced the development of two-photon optogenetic stimulation methods as a trajectory toward the goal of applying 3D-SHOT to large FOV two-photon microscopy. The field progressed from early proof-of-principle studies using spiral scanning to the adoption of temporal focusing and holography and eventually to their combination for three-dimensional implementations. In practice, however, the approach most commonly chosen and successfully applied in neuroscience experiments at present is to generate two- or three-dimensional holograms with an SLM and scan them with galvanometric mirrors.

The fundamental distinction between this method and 3D-SHOT lies in whether temporal focusing or galvanometric scanning is used. In terms of stimulation speed, 3D-SHOT is advantageous, as opsins distributed across the membrane can be activated simultaneously, whereas spiral scanning is inherently limited by the millisecond-scale response time of the galvos. By contrast, laser power requirements favor the hologram-plus-spiral approach: by generating diffraction-limited holographic foci and scanning them across a soma, it is possible to achieve activation with lower average power. In 3D-SHOT, however, the light intensity must reach two-photon excitation thresholds over an entire soma-sized (or larger) volume, which demands substantially higher power. As a result, compared with holographic spiral scanning, 3D-SHOT offers lower latency and jitter, but at the cost of requiring high-power lasers and posing an increased risk of tissue heating. The potential for tissue damage due to elevated laser power is a serious concern.

Taken together, holographic spiral scanning remains the more practical and widely used approach at present. If future technical advances resolve the power requirement, however, 3D-SHOT may become a more suitable method.

Another important point is that although two-photon optogenetic stimulation represents an optimal tool for manipulating the activity of precisely defined neuronal ensembles, such technically elaborate experiments are not always necessary, depending on the experimental aim. For example, in our previous work,^46^ opsins were sparsely expressed in ∼5% of neurons in the motor cortex. One-photon stimulation was applied during two-photon calcium imaging, whereas water rewards were delivered either before or after photostimulation. Within only 15 min of training, mice increased the spontaneous activity of stimulated neurons when stimulation preceded reward, but decreased their activity when reward preceded stimulation. This bidirectional modulation may represent a fundamental mechanism underlying the association between actions and rewards.

Similarly, in the study by Carrillo-Reid et al.,^47^ opsins were expressed in visual cortical neurons, and a two-photon stimulation laser was scanned across the entire imaging field at specific time points to activate all opsin-expressing neurons within view. Repeated stimulation imprinted a functional ensemble independent of visual input, which maintained its functional connectivity across days. Strikingly, stimulation of a single neuron within this ensemble was sufficient to reactivate the entire ensemble. The authors referred to this process as pattern completion and the reactivation as recall.

These findings demonstrate that meaningful manipulations of ensemble activity, and their behavioral consequences, can often be achieved with simpler strategies—such as sparse opsin expression combined with one-photon stimulation, or large FOV two-photon scanning—despite their reduced specificity and flexibility compared with advanced approaches such as 3D-SHOT. Thus, experimenters should avoid being overly captivated by technically “fancy” methods, and instead design experiments in a purpose-driven manner aligned with the scientific question at hand.

Although this article has emphasized optical methodologies, it is also worth briefly summarizing what has been learned from two-photon optogenetic stimulation itself. A simple yet striking finding across many studies is that photostimulation of excitatory neurons generally produced net suppressive effects in local circuits. This suppressive influence was observed both in the immediate neighborhood of the stimulated cells and in distant cortical regions. These results clearly indicate that cortical circuits maintain a finely tuned balance between excitation and inhibition, such that additional excitatory drive engages inhibitory interneurons and normalizes the overall spike output to approximately zero net change.

Nevertheless, several studies have reported pattern completion, whereby activation of a subset of neurons can trigger activity across an entire functional ensemble. This suggests that although the overall circuit response tends to be suppressive, functionally related subsets of neurons can still be strongly activated. Such observations are consistent with the known anatomical organization of local cortical networks.^48^

By contrast, the behavioral consequences of stimulation appear to vary substantially with context and thus require careful re-examination under diverse conditions and brain states before firm conclusions can be drawn. Given the inherent complexity of the brain, the effects of stimulation in specific cognitive tasks or contexts may lead to different interpretations depending on subtle differences in experimental design. These technically demanding experiments therefore require not only cautious interpretation of results but also close evaluation of the methodological assumptions underlying them.

## 4 Related Techniques and Outstanding Issues

In this section, we briefly summarize additional technical advances and challenges in two-photon optogenetic stimulation that were not covered in the previous discussion. In 3D-SHOT, because the disk-shaped pattern at the entrance of the diffraction grating is replicated by the SLM and distributed in three dimensions, the freedom to control spot size and shape under the objective lens is limited. To address this, Accanto et al.^49^ introduced a second SLM before the grating, enabling arbitrary control over the input shape. This approach, termed multiplexed temporally focused CGH, successfully enhanced the flexibility of optogenetic stimulation in terms of spot size and geometry.

Similarly, Faini et al.^50^ proposed a strategy called *FLiT*, in which a galvanometric mirror is placed upstream of the SLM to divide the SLM surface into multiple regions. Although updating an SLM phase pattern typically requires several milliseconds, this configuration allows rapid selection of preloaded holograms with the galvo, achieving sub-millisecond updates of stimulation patterns.

Inazawa et al.^51^ employed an echelle grating to introduce optical path differences larger than the pulse width in the direction orthogonal to spectral dispersion. In combination with a DMD that generated multiple line segments, fast and accurate acquisition of optical sectioning was achieved (time-multiplexed multifocal temporal focusing). Ishikawa et al.^52^ extended this by adding an SLM, applying CGH to suppress interference fringes and speckle, a method that should also be applicable to two-photon stimulation.

Although galvanometric mirrors require milliseconds to switch stimulation among individual cells, so-called random-access scanning with acousto-optic deflectors (AODs) can reduce this timescale to the microsecond range. This is faster than the intrinsic timescale of neuronal ensemble dynamics, making AOD-based approaches promising for two-photon optogenetic stimulation with diffraction-limited beams. Indeed, AODs are already widely used in fast two-photon imaging.^53^^–^^56^ However, no reports have yet demonstrated large-scale three-dimensional two-photon optogenetic stimulation using AODs. This may reflect fundamental limitations: because AODs create grating-like patterns using ultrasound, different wavelengths become spatially separated, leading to pulse broadening; moreover, the scanning angle is inherently restricted. Nevertheless, the commercial availability of the FEMTO3D Atlas (Femtonics, Budapest, Hungary), which supports AOD-based two-photon optogenetics, indicates that the technology has reached sufficient stability for routine use, and it may become more widely adopted in the near future.

We next turn to algorithms for generating CGH. In most studies, the phase masks displayed on the SLM are computed using the GS algorithm.^21^ The GS algorithm iteratively optimizes the SLM phase pattern by alternately applying Fourier and inverse Fourier transforms between the Fourier plane, where the SLM is located, and the focal plane. As SLMs generally modulate only the phase, the intensity distribution of the incident coherent laser beam directly constrains the reflected intensity distribution, which imposes a boundary condition on the optimization. A common issue is that naïve implementations produce weaker illumination at the periphery of the field relative to the center. To compensate for this, preprocessing steps—such as modifying the target intensity pattern so that its periphery is artificially brightened—are often applied.

Beyond GS, alternative algorithms have been developed. NOVO-CGH^36^ directly optimizes a two-dimensional phase mask from a desired three-dimensional intensity distribution. More recently, Deep-CGH^57^ employs convolutional neural networks (CNNs) to directly predict SLM phase masks from target intensity patterns. Because Deep-CGH can generalize once trained on a sufficiently large dataset of input–output pairs, and because feedforward prediction is substantially faster than iterative GS or NOVO-CGH optimization, this approach has the potential to become the dominant strategy in future applications.

To briefly summarize the opsins commonly used for two-photon optogenetic stimulation, among excitatory variants, *ChRmine*^24^ exhibits the largest photocurrents and highest light sensitivity, its red-shifted derivative *rsChRmine*^58^ minimizes crosstalk with imaging lasers, *Chronos*^59^ produces similarly large currents but with faster kinetics than ChRmine, *ChroME*^12^ is a modified form of Chronos, and *ChroME2s*^37^ is a further engineered variant with enhanced performance. For inhibitory control, *GtACR1*^60^ and its modified form *eGtACR1*^17^ are frequently employed. More recently, potassium-selective opsins that are excitable by two-photon illumination^61^^,^^62^ have been developed, representing a promising direction for future research. In most cases, these opsins are targeted to the soma by inclusion of the Kv2.1 trafficking sequence.

It is well recognized that achieving balanced co-expression of opsins and calcium indicators in the same neurons via viral vectors is technically challenging. To overcome this, genetically engineered mouse lines such as Ai203 (TITL-st-ChroME-GCaMP7s-ICL-nls-mRuby3-IRES2-tTA2),^45^ which enable stable co-expression of opsins and GCaMP, have been developed. Similar lines may become the new standard for two-photon all-optical physiology in the near future.

Several recent studies have demonstrated the feasibility of two-photon optogenetic stimulation in deep brain structures. Vinograd et al.^30^ perturbed attractor dynamics in the hypothalamus that encode emotional states and examined their stability. They used *Kv2.1-ChRmine* as the opsin and the NeuraLight 3D system (Bruker) for two-photon stimulation. The stimulation laser was a Monaco-1035-40-40 (1 MHz, 40 W, 300 fs), and the SLM was a 512×512 device, which is likely the standard configuration of the NeuraLight 3D.

Piantadosi et al.^29^ investigated the basolateral amygdala, identifying two neuronal populations associated with appetitive and aversive emotional behaviors. Selective stimulation of one ensemble suppressed behavior associated with the other. They also employed *Kv2.1-ChRmine* with a Bruker two-photon system, most likely the NeuraLight 3D given the reported 512×512 SLM. The stimulation laser was a Spirit One (1040 nm, ∼8  W, 1 MHz, ∼500  fs).

To this point, all implementations have been restricted to head-fixed animals. However, head fixation is a significant limitation for studying naturalistic behavior. A system termed 2P-FENDO^63^ enables the combined use of two-photon imaging and two-photon optogenetic stimulation in freely moving mice. Opsin expression relied on *Kv2.1-ChRmine*. The stimulation source was a Goji laser (Amplitude; 10 MHz, 8 W, 150 fs). The SLM was a Hamamatsu X10468-07 (792×600  pixels, 20-μm pitch, 15.8  mm×12  mm, rise time 10 ms, fall time 80 ms), positioned at a Fourier plane. The distal end of a fiber bundle and the focal planes of GRIN lenses on both sides were conjugated to the same focal plane. Each fiber in the bundle (FIGH-15-600N, Fujikura) differs in optical path length by ∼0.1  mm per meter, introducing relative delays on the order of 1 picosecond among fibers. Consequently, although a 100-fs pulse is preserved within a single fiber, output from different fibers does not recombine temporally. Instead, the SLM generates a two-dimensional hologram corresponding to the physical arrangement of fibers, each mapped to the spatial position of target neurons. Because there is no crosstalk among fibers, this two-dimensional (2D) holographic pattern is faithfully transferred to the GRIN lens focal plane. For example, if a single neuron corresponds to ∼3 fibers, each laser pulse delivers three temporally separated stimuli to the same cell [Fig. 2(e)]. It is likely that the fiber dimensions were deliberately matched to the size of target neurons to optimize spatial resolution.

At the end of this section, we briefly discuss the problem of crosstalk between two-photon optogenetics and two-photon calcium imaging, as well as possible solutions. Crosstalk arises when the action spectra of the opsin and the fluorescent indicator overlap, such that the imaging laser unintentionally activates the opsin. The opposite situation, in which photostimulation interferes with calcium imaging, can also be problematic, especially in closed-loop experiments or BMI settings where stimulation occurs continuously during imaging.

Regarding the first problem, how can the unintended activation of the opsin by the imaging laser be avoided? Three main strategies are available. First, one can use or engineer opsins that are minimally activated at 920 nm, the wavelength typically used for GCaMP imaging. Opsins such as C1V1,^11^ ChrimsonR,^12^ and ChRmine^24^^,^^58^ have action spectra that are red-shifted into the 1040 to 1100 nm range, making them relatively suitable for this purpose, although not perfect. Alternatively, it is also possible to perform imaging at longer wavelengths while delivering photostimulation at shorter wavelengths, and several studies have reported the combination of jRCaMP1a (imaged at 1040 to 1100 nm) with stCoChR or GtACR2 (stimulated at 920 nm).^64^^,^^65^ Although each implementation requires optimization of experimental parameters, further genetic engineering to increase wavelength specificity for both stimulation and imaging will be desirable to enhance the general applicability of this approach in the future. Second, opsin expression can be restricted to the soma using targeting motifs such as Kv2.1.^13^^,^^66^ Soma-restricted expression minimizes the influence of the imaging laser on non-targeted neurites and reduces unintended opsin activation outside the soma. Third, the neuronal populations used for stimulation and those used for imaging can be spatially separated. With large field-of-view two-photon microscopy, this becomes practically feasible because imaging and stimulation planes or regions can be allocated to distinct neuronal ensembles.^5^

We now consider the opposite case, in which photostimulation interferes with imaging. In general, GCaMP exhibits only weak two-photon excitation at 1040 nm, so this combination is usually not a major problem. In cases where photostimulation does cause interference, if the stimulation is temporally sparse, its effects can be mitigated by excluding those time points from analysis, turning off the photomultiplier, or using shutter control during stimulation.

## 5 Applying Multiphoton Techniques to BMI

Finally, we highlight BMIs as an application that lies along the trajectory of two-photon optogenetics. BMIs map neural activity to the control of external devices (e.g., robotic arms or computer cursors), directly translating a subject’s intentions into actions; they thus constitute a clinically important avenue with potential to improve the quality of life of patients with ALS or severe paralysis. Traditionally, neural activity has been recorded electrophysiologically, but single-cell recordings require invasive electrode implantation, and electrical stimulation co-activates passing axons, limiting spatial specificity.^67^ Optical approaches offer a promising alternative.^46^^,^^68^^,^^69^ Two-photon calcium imaging far exceeds electrophysiology in the number of simultaneously recorded neurons, and two-photon optogenetic stimulation remains the only method for precisely manipulating targeted ensembles. Here, we briefly outline examples of BMIs implemented with two-photon microscopy and sketch future directions informed by two-photon optogenetics. Although some issues remain at the level of thought experiments, these technical challenges represent reasonable near-term goals for optics, electronics, and molecular biology; for a comprehensive discussion, see our previous review article.^69^ For general information on BMI, there are excellent review articles available, and we encourage readers to refer to those.^70^^–^^75^

### 5.1 Two-Photon BMIs: Examples

The basic principle of BMI is to map neural signals from awake, behaving brains onto external actions; when the resulting outcomes are beneficial, subjects can adaptively modulate their brain activity. The central technical challenge is real-time readout. Under two-photon imaging, this requires integrating acquisition with online analysis and feedback. We built such a BMI in the mouse primary/secondary motor cortex: using two-photon calcium imaging, we analyzed the activity of a single neuron in real time and coupled it to reward. Mice significantly increased the activity of the target cell within 15 min,^46^ indicating that reinforcement or attenuation of cortical activity synchronized to reward timing can be reproduced even under artificial manipulation. Furthermore, Mitani et al.^76^ developed a two-photon BMI targeting inhibitory cell types (PV, SOM, and VIP) and demonstrated cell-type-dependent differences in controllability. In nonhuman primates, Trautmann et al.^77^ monitored dendritic activity in macaque cortex with two-photon calcium imaging and successfully decoded movement intention in real time—an important step toward human application.

Taken together, BMIs based on two-photon microscopy already have a history spanning more than a decade, and, technically, extension to humans is increasingly within reach.

### 5.2 Potential BMIs Based on Optical Recording

Below, we consider a scenario in which two-photon microscopy propels BMI technology beyond electrophysiological measurement-and-control frameworks. To date, two-photon BMIs—including our own—have operated external devices with the activity of at most a few neurons. This scale can readily be expanded to hundreds, and, with large FOV two-photon microscopy, tens of thousands of neurons can be recorded over months.^41^ Thus, an increase in throughput by $∼10^{4}\times$ is already within reach. Learning has been shown to accelerate when two-photon BMI is paired with simultaneous one-photon photostimulation feedback^78^; replacing this with two-photon optogenetic stimulation of targeted ensembles should further improve efficiency. Closed-loop experiments in which two-photon optogenetic stimulation is adjusted in real time based on two-photon calcium imaging have also been reported.^79^ To stimulate novel ensembles in real time and in parallel, SLM phase masks must be generated on the fly; current SLMs can update within ≤2  ms, and DeepCGH can compute phase patterns on comparable timescales.^57^ As reviewed here, the scale of simultaneous two-photon stimulation looks promising with 3D-SHOT^17^^,^^35^ and its adaptation to large FOV systems.^26^ Real-time image processing and high-speed analysis of large imaging streams during BMI operation are technically tractable using FPGAs, GPUs, or both.^80^ For use away from the bedside, miniaturization of two-photon microscopes will be necessary; apart from the laser, significant progress has already been made.^63^

In addition, optical methods now allow accurate *in vivo* measurement of neuromodulators including dopamine, acetylcholine, noradrenaline, serotonin, histamine, and opioids and, in some cases, optical control of their concentrations.^81^ Human translation of optogenetics will require safer viral vector strategies, but progress is evident in studies of the retina and diseased human brain tissue,^82^^–^^84^ as well as in viruses capable of crossing the blood–brain barrier.^85^^,^^86^ Given the maturity of electrophysiology, transparent electrodes that permit the coexistence of optical and electrical interfaces will be valuable.^87^ Because interventions will be highly invasive, the ethical dimensions require careful consideration.^88^

Taken together, leveraging the fundamental advantages of optical measurement and control and coupling multimodal readouts with single-cell-resolution control in real-time closed loop should enable flexible, multifunctional, and task-general optical BMIs.

### 5.3 Future BMI Based on Two-Photon Optogenetics

Building on the technological achievements discussed in the previous sections, we consider what kinds of BMIs could become possible when two-photon optogenetic stimulation is incorporated. Two-photon feedback stimulation has the potential to go far beyond conventional BMIs and to enable entirely new concepts, including the following:

1. whole-body BMIs with artificial proprioception
2. representation sculpting for fine-grained neural control
3. acceleration of computational resource transfer during learning
4. bidirectional interfaces between the brain and external AI models.

Together, these directions represent a major step toward BMIs that can directly read from and write into the brain’s representational space, something that has been difficult to achieve with electrophysiology-based BMIs. We detail each of these concepts below.

#### 5.3.1 Whole-body BMIs enabled by artificial proprioception

Two-photon stimulation makes it possible to provide artificial somatosensory and proprioceptive feedback that does not rely on visual feedback. Traditional BMIs depend almost exclusively on visual feedback, and the absence of proprioceptive signals provided by muscle spindles and joint receptors has been a major limitation for both learning efficiency and control precision. By delivering selective two-photon optogenetic stimulation to primary or higher-order somatosensory cortices, it becomes possible to generate “artificial proprioceptive signals” corresponding to multiple body parts—such as the hand, shoulder, trunk, or legs—at single-neuron resolution. This capability provides a critical path toward expanding BMIs from single-joint or cursor-based systems to integrated whole-body BMIs.

#### 5.3.2 Representation sculpting for fine-grained neural control

Two-photon stimulation also enables the sculpting of neural representations. Two-photon imaging can identify cell populations that encode specific body parts such as the hand, shoulder, or trunk. Ideally, these populations would be independently controllable. However, correlations among such neural populations can cause interference. For example, attempting to move the left hand may inadvertently recruit representations related to the right hand. With conventional BMIs, resolving such interference is left entirely to the participant’s learning. In contrast, two-photon stimulation may allow us to intentionally decorrelate these representations. By doing so, neural population activity corresponding to intended versus unintended movements could be sufficiently separated and stabilized along independent dimensions. This would allow the formation of distinct control dimensions within the brain, improving decoding accuracy and enabling BMIs capable of more complex and structured behaviors.

#### 5.3.3 Acceleration of computational resource transfer during learning

Two-photon stimulation may further facilitate the hierarchical allocation of computational resources during learning. In motor learning, flexible representations in higher-order motor areas play a central role during exploration, whereas with practice, control becomes consolidated into lower-order regions such as the primary motor cortex or alternatively into the cerebellum or basal ganglia. By selectively building up activity in lower-order regions using two-photon stimulation, it may be possible to accelerate this natural transition of representation from higher to lower areas, thereby promoting faster and more physiologically aligned BMI learning. This approach offers advantages for both learning speed and stability of control. Furthermore, if higher-order regions can be trained to express more abstract intentions, hierarchical and complex behaviors may become achievable through BMI control.

#### 5.3.4 Direct coupling of brain representations with external models

Finally, the ability to manipulate specific neural representations online with two-photon stimulation opens the possibility of using external recurrent neural network (RNNs) or transformers as extended representational systems for the brain [Fig. 1(e)]. By accessing the state of an external model and incorporating it into its own representations via stimulation, the subject may gain indirect access to external memory, external reasoning modules, or even information available on the web. In other words, optical BMIs have the potential to function as “extended cognitive interfaces” that directly couple the brain with artificial models at the level of neural population activity.

### 5.4 Required Precision of Two-Photon Optogenetic Stimulation for BMI Applications

Finally, we consider what is currently achievable with existing technology and what level of advancement is required for the next steps, focusing in particular on the number of neurons that can be stimulated simultaneously and the temporal resolution needed.

To accomplish tasks, Secs. 5.3.1 and 5.3.2 described in Sec. 5.3, at least several hundred neurons within a given cortical area must be brought under controllable range. This level of control has already been demonstrated with 3D-SHOT.^17^^,^^35^ If this capability can be extended across multiple brain areas, integrating 3D-SHOT into a large field-of-view two-photon microscope may enable the realization of task Sec. 5.3.3. Further expanding control to several thousand neurons, or even more across multiple cortical areas, would bring task Sec. 5.3.4 within reach, although current laser power limitations make it difficult to control more than several thousand neurons. Although achieving large-scale control immediately is not easy, proof-of-concept experiments at smaller scales are already feasible with existing photostimulation technologies. However, sustained illumination of the brain with high laser power can induce cytotoxicity due to tissue heating, and therefore it will also be necessary to develop approaches that use optical energy more efficiently.

With regard to temporal resolution, it is essential to consider the timescale at which the expected neural or behavioral effects emerge following stimulation. The brain implements learning rules such as unsupervised learning, supervised learning, and reinforcement learning, which operate on characteristic timescales of ∼10  ms, 100 ms, and 1 s.^69^ In two-photon photostimulation, the typical latency from stimulation to spike generation generally satisfies a temporal resolution of several tens of milliseconds. Although higher temporal precision is desirable, it competes with the number of neurons that can be stimulated simultaneously. Therefore, these broad learning timescales provide a useful guideline for determining the required temporal resolution of two-photon photostimulation depending on the intended BMI application.

Overall, the key enabling technologies are already in place, and the groundwork for realizing these ideas is largely prepared. Further improvements in fundamental components, including more sensitive opsins, optimization of excitation wavelengths, and enhanced laser performance, will become increasingly important for expanding the achievable scale of neural control for the BMI.

### 5.5 Future BMI Based on Two-Photon Optogenetics Targeting Synapse

In the preceding sections, we considered the incorporation of two-photon optogenetic stimulation into BMIs at the cellular level. Here, we explore what might become possible if two-photon optogenetic stimulation could be performed at the level of individual synapses. Various approaches for synaptic optogenetics have already been proposed.^89^ The critical difference from manipulating neural activity at the cellular level is that controlling synaptic plasticity allows direct modification of the circuit itself.

In CNNs and RNNs, synaptic plasticity that optimizes a specific objective function can be implemented using backpropagation. If an analogous mechanism could be applied to the brain, it might, in principle, allow the acquisition of arbitrary functions. In other words, by appropriately controlling synaptic plasticity, one could optimize any desired objective function or computational capability.

However, the central challenge lies in identifying in advance which synapses influence which aspects of BMI control and in what manner. This is essentially the synaptic credit assignment problem. In artificial neural networks, all synaptic weights and activation functions are explicitly known, enabling differentiation of the objective function and propagation of error signals down the hierarchy to determine each synapse’s contribution. By contrast, the brain’s connectivity and nonlinearities are not explicitly accessible.

If it becomes possible to measure tens of thousands of neurons and large numbers of synaptic events simultaneously, and to model how each synapse contributes to decoding accuracy or actuator control, it may become feasible to predict how strengthening a particular group of spines would alter circuit-wide dynamics and BMI performance. Based on such predictions, selectively strengthening or weakening only the most influential spines through two-photon optogenetic stimulation could allow targeted functional write-in at the circuit level. In other words, synaptic intervention would require measurements that are sufficiently large-scale and precise to predict which spines should be modified.

Nevertheless, credit assignment remains difficult even with advanced measurement technologies, especially when the influence of a given synapse propagates across many synapses or becomes embedded in complex network dynamics. Fundamentally, we still do not understand how the brain itself resolves this problem. Continued basic research is needed to clarify how the brain uses synaptic plasticity to solve tasks.

Taken together, the most realistic application may be to use synaptic-level interventions as support for the brain’s intrinsic circuit plasticity. Synapses that are important for solving a given task will naturally begin to undergo plasticity. By delivering stimulation that supports the maturation of this emerging plasticity, it may be possible to accelerate learning. This approach assists the brain’s own problem-solving mechanisms without requiring explicit knowledge of how the brain solves the task, and in this sense, leverages the brain’s inherent computational capabilities.

### 5.6 Software for Two-Photon BMI and Two-Photon Optogenetic Stimulation

In this final subsection, we summarize representative algorithms and software that can be used for the applications described above. We highlight three categories that are especially relevant for *in vivo* two-photon BMI:

1. real-time imaging analysis [motion correction and region of interest (ROI) extraction].
2. latent-state inference for neural decoding and closed-loop behavioral control.
3. CGH algorithms and SLM control.

#### 5.6.1 Real-time imaging analysis (motion correction and ROI extraction)

Representative real-time imaging analysis frameworks include Suite2p, CaImAn, ORCA, and NeuroART. Suite2p^90^ provides a high-speed pipeline implementing real-time motion correction, automated cell detection, deltaF/F extraction, and spike inference for large-scale calcium imaging datasets. Under GPU acceleration, its processing speed exceeds typical image acquisition rates, and Suite2p is widely used for online monitoring of spontaneous and stimulus-driven activity in head-fixed mice. CaImAn^91^ supports an online mode that performs sequential drift correction using the NoRMCorre algorithm,^92^ along with real-time cell extraction and spike reconstruction based on CNMF.^93^ This makes closed-loop experiments feasible during simultaneous behavioral monitoring and neural recording. NeuroART^94^ is designed to detect and target neuronal populations exhibiting stimulus-driven modulation in real time, providing a framework that explicitly links online neural-population analysis with optogenetic manipulation. ORCA^95^ similarly offers an integrated environment for real-time imaging analysis, enabling acquisition of single-neuron deltaF/F signals while dynamically adjusting photostimulation based on ongoing neural activity.

#### 5.6.2 Latent-state inference for decoding and behavioral control

We next describe algorithms and software for decoding neural population activity from two-photon calcium imaging data. Several latent-variable models have been developed specifically for handling calcium dynamics. RADICaL^96^ extends the AutoLFADS^97^^,^^98^ variational framework to infer high-frequency latent activity vectors from calcium event sequences. VaLPACa (Prince et al., 2021)^99^ models calcium signals as a mixture of calcium flux and underlying dynamics and uses hierarchical variational inference to disentangle these components. This enables more accurate extraction of neural population dynamics by explicitly accounting for calcium time constants and noise. In addition, methods based on tensor component analysis (TCA) and its variants such as sliceTCA can efficiently extract low-dimensional single-trial neural dynamics from large-scale population activity, providing compact representations that are well suited for subsequent decoding and modeling.^100^^,^^101^ Although these algorithms do not operate online, once latent representations are extracted, real-time decoding becomes straightforward. Combining such latent-state models with real-time imaging pipelines will likely facilitate more efficient extraction of neural representations for BMI control.

Linking neural decoding to behavior requires real-time behavioral tracking. DeepLabCut^102^ provides markerless tracking of body parts using deep learning, and DLC-Live^103^ achieves low-latency posture estimation and real-time feedback at ∼15  ms. Real-time alignment of latent neural states with behavioral variables is an essential step for advancing two-photon BMIs.

#### 5.6.3 CGH algorithms and SLM control

For two-photon holographic stimulation, CGH algorithms are central because they compute the phase mask that converts the desired illumination pattern into SLM control signals. The classical GS algorithm remains widely used due to its simplicity and is implemented in many SLM control platforms, including ScanImage. DeepCGH^57^ is a learning-based framework that employs convolutional neural networks to directly generate phase masks from target intensity patterns. This approach enables orders-of-magnitude faster computation and higher-fidelity reconstructions compared with traditional iterative methods, making it particularly suitable for real-time or large-scale holographic stimulation.

## 6 Conclusion

Focusing on two-photon optogenetic stimulation, we have surveyed a set of optical techniques that continue to evolve. We identified two major directions for activating opsins via two-photon absorption: (i) shaping the excitation pattern to match cellular morphology and (ii) employing low-repetition-rate femtosecond pulsed lasers. The first direction has been realized through spiral scanning, temporal focusing, and CGH. At present, a widely adopted strategy is to generate, at the sample plane under the objective, a hologram smaller than a neuron and rotate it in a spiral with galvanometric mirrors. We also note that 3D-SHOT—combining holography with temporal focusing—currently holds the record for the largest number of cells activated simultaneously. These approaches are being applied with optical fibers and GRIN lenses, as well as in large two-photon microscopes such as 2P-RAM and Diesel2p, and can be integrated into closed-loop experimental systems to advance toward BMIs. Our team is pursuing this trajectory, aiming to expand the bidirectional communication bandwidth between the brain and AI systems. Although the timing of clinical translation remains uncertain, we have outlined specific technical challenges that must be addressed. Taken together, advances in these component technologies and their combinations point toward a future that, whereas once reminiscent of science fiction, is becoming technically attainable. In parallel with progress in AI, the continued development of these methods will have significance that extends beyond basic neuroscience.

## Data Availability

Data sharing is not applicable to this article, as no new data were created or analyzed.

## References

[1] Hira R. et al., “Spatiotemporal dynamics of functional clusters of neurons in the mouse motor cortex during a voluntary movement,” J. Neurosci. 33(4), 1377–1390 (2013). JNRSDS 0270-6474 10.1523/JNEUROSCI.2550-12.2013 23345214 PMC6618743

[2] Nakai J. Ohkura M. Imoto K., “A high signal-to-noise ${\mathrm{Ca}}^{2+}$ probe composed of a single green fluorescent protein,” Nat. Biotechnol. 19(2), 137–141 (2001). NABIF9 1087-0156 10.1038/84397 11175727

[3] Zhang Y. et al., “Fast and sensitive GCaMP calcium indicators for imaging neural populations,” Nature 615(7954), 884–891 (2023). 10.1038/s41586-023-05828-9 36922596 PMC10060165

[4] Caporale N. Dan Y., “Spike timing–dependent plasticity: a Hebbian learning rule,” Annu. Rev. Neurosci. 31(1), 25–46 (2008). ARNSD5 0147-006X 10.1146/annurev.neuro.31.060407.125639 18275283

[5] Abdeladim L. et al., “Probing inter-areal computations with a cellular resolution two-photon holographic mesoscope,” bioRxiv (2023). 10.1038/s41593-026-02350-9 PMC13433287 42547821

[6] Nagel G. et al., “Channelrhodopsin-2, a directly light-gated cation-selective membrane channel,” Proc. Natl. Acad. Sci. U. S. A. 100(24), 13940–13945 (2003). 10.1073/pnas.1936192100 14615590 PMC283525

[7] Rickgauer J. P. Tank D. W., “Two-photon excitation of channelrhodopsin-2 at saturation,” Proc. Natl. Acad. Sci. U. S. A. 106(35), 15025–15030 (2009). 10.1073/pnas.0907084106 19706471 PMC2736443

[8] Packer A. M. et al., “Two-photon optogenetics of dendritic spines and neural circuits,” Nat. Methods 9(12), 1202–1205 (2012).1548-7091 10.1038/nmeth.2249 23142873 PMC3518588

[9] Prakash R. et al., “Two-photon optogenetic toolbox for fast inhibition, excitation and bistable modulation,” Nat. Methods 9(12), 1171–1179 (2012).1548-7091 10.1038/nmeth.2215 23169303 PMC5734860

[10] Chettih S. N. Harvey C. D., “Single-neuron perturbations reveal feature-specific competition in V1,” Nature 567(7748), 334–340 (2019). 10.1038/s41586-019-0997-6 30842660 PMC6682407

[11] Yizhar O. et al., “Neocortical excitation/inhibition balance in information processing and social dysfunction,” Nature 477(7363), 171–178 (2011). 10.1038/nature10360 21796121 PMC4155501

[12] Klapoetke N. C. et al., “Independent optical excitation of distinct neural populations,” Nat. Methods 11(3), 338–346 (2014).1548-7091 10.1038/nmeth.2836 24509633 PMC3943671

[13] Baker C. A. et al., “Cellular resolution circuit mapping with temporal-focused excitation of soma-targeted channelrhodopsin,” eLife 5, e14193 (2016). 10.7554/eLife.14193 27525487 PMC5001837

[14] Oron D. Tal E. Silberberg Y., “Scanningless depth-resolved microscopy,” Opt. Express 13(5), 1468–1476 (2005). OPEXFF 1094-4087 10.1364/OPEX.13.001468 19495022

[15] Andrasfalvy B. K. et al., “Two-photon single-cell optogenetic control of neuronal activity by sculpted-light,” Proc. Natl. Acad. Sci. U. S. A. 107(26), 11981–11986 (2010). 10.1073/pnas.1006620107 20543137 PMC2900666

[16] Papagiakoumou E. Ronzitti E. Emiliani V., “Scanless two-photon excitation with temporal focusing,” Nat. Methods 17(6), 571–581 (2020).1548-7091 10.1038/s41592-020-0795-y 32284609

[17] Mardinly A. R. et al., “Precise multimodal optical control of neural ensemble activity,” Nat. Neurosci. 21(6), 881–893 (2018). NANEFN 1097-6256 10.1038/s41593-018-0139-8 29713079 PMC5970968

[18] Rickgauer J. P. Deisseroth K. Tank D. W., “Simultaneous cellular-resolution optical perturbation and imaging of place cell firing fields,” Nat. Neurosci. 17(12), 1816–1824 (2014). NANEFN 1097-6256 10.1038/nn.3866 25402854 PMC4459599

[19] Nikolenko V. et al., “SLM microscopy: scanless two-photon imaging and photostimulation using spatial light modulators,” Front. Neural Circuits 2, 393 (2008). 10.3389/neuro.04.005.2008 PMC2614319 19129923

[20] Packer A. M. et al., “Simultaneous all-optical manipulation and recording of neural circuit activity with cellular resolution in vivo,” Nat. Methods 12(2), 140–146 (2015).1548-7091 10.1038/nmeth.3217 25532138 PMC4933203

[21] Gerchberg R. W., “A practical algorithm for the determination of plane from image and diffraction pictures,” Optik 35(2), 237–246 (1972). OTIKAJ 0030-4026

[22] Yang W. et al., “Simultaneous two-photon imaging and two-photon optogenetics of cortical circuits in three dimensions,” eLife 7, e32671 (2018). 10.7554/eLife.32671 29412138 PMC5832414

[23] Carrillo-Reid L. et al., “Controlling visually guided behavior by holographic recalling of cortical ensembles,” Cell 178(2), 447–457.e5 (2019). CELLB5 0092-8674 10.1016/j.cell.2019.05.045 31257030 PMC6747687

[24] Marshel J. H. et al., “Cortical layer–specific critical dynamics triggering perception,” Science 365(6453), eaaw5202 (2019). SCIEAS 0036-8075 10.1126/science.aaw5202 31320556 PMC6711485

[25] Dalgleish H. W. et al., “How many neurons are sufficient for perception of cortical activity?” eLife 9, e58889 (2020). 10.7554/eLife.58889 33103656 PMC7695456

[26] Fişek M. et al., “Cortico-cortical feedback engages active dendrites in visual cortex,” Nature 617(7962), 769–776 (2023). 10.1038/s41586-023-06007-6 37138089 PMC10244179

[27] Russell L. E. et al., “The influence of cortical activity on perception depends on behavioral state and sensory context,” Nat. Commun. 15(1), 2456 (2024). NCAOBW 2041-1723 10.1038/s41467-024-46484-5 38503769 PMC10951313

[28] Wu A. et al., “Targeted stimulation of motor cortex neural ensembles drives learned movements,” bioRxiv (2025).

[29] Piantadosi S. C. et al., “Holographic stimulation of opposing amygdala ensembles bidirectionally modulates valence-specific behavior via mutual inhibition,” Neuron 112(4), 593–610.e5 (2024). NERNET 0896-6273 10.1016/j.neuron.2023.11.007 38086375 PMC10984369

[30] Vinograd A. et al., “Causal evidence of a line attractor encoding an affective state,” Nature 634(8035), 910–918 (2024). 10.1038/s41586-024-07915-x 39142337 PMC11499281

[31] Papagiakoumou E. et al., “Scanless two-photon excitation of channelrhodopsin-2,” Nat. Methods 7(10), 848–854 (2010).1548-7091 10.1038/nmeth.1505 20852649 PMC7645960

[r32] Papagiakoumou E. et al., “Temporal focusing with spatially modulated excitation,” Opt. Express 17(7), 5391–5401 (2009). OPEXFF 1094-4087 10.1364/OE.17.005391 19333304

[33] Papagiakoumou E. et al., “Patterned two-photon illumination by spatiotemporal shaping of ultrashort pulses,” Opt. Express 16(26), 22039–22047 (2008). OPEXFF 1094-4087 10.1364/OE.16.022039 19104638

[34] Chen I. W. et al., “In vivo submillisecond two-photon optogenetics with temporally focused patterned light,” J. Neurosci. 39(18), 3484–3495 (2019). JNRSDS 0270-6474 10.1523/JNEUROSCI.1785-18.2018 30833505 PMC6495136

[35] Pégard N. C. et al., “Three-dimensional scanless holographic optogenetics with temporal focusing (3D-SHOT),” Nat. Commun. 8(1), 1228 (2017). NCAOBW 2041-1723 10.1038/s41467-017-01031-3 29089483 PMC5663714

[36] Zhang J. et al., “3D computer-generated holography by non-convex optimization,” Optica 4(10), 1306–1313 (2017). 10.1364/OPTICA.4.001306

[37] Sridharan S. et al., “High-performance microbial opsins for spatially and temporally precise perturbations of large neuronal networks,” Neuron 110(7), 1139–1155.e6 (2022). NERNET 0896-6273 10.1016/j.neuron.2022.01.008 35120626 PMC8989680

[38] Oldenburg I. A. et al., “The logic of recurrent circuits in the primary visual cortex,” Nat. Neurosci. 27(1), 137–147 (2024). NANEFN 1097-6256 10.1038/s41593-023-01510-5 38172437 PMC10774145

[39] Bounds H. A. Adesnik H., “Network influence determines the impact of cortical ensembles on stimulus detection,” Neuron, 113(14), 2358–2369 (2024). 10.1101/2024.08.18.608496 PMC12934253 40378835

[40] Hira R. et al., “Open-source modular FPGA system for two-photon mesoscope enabling multi-layer, multi-depth neural activity recording and lifetime imaging,” bioRxiv (2025). 10.1117/1.NPh.13.1.015013 PMC12956299 41782611

[r41] Hira R. et al., Mesoscale Functional Architecture in Medial Posterior Parietal Cortex, eLife Sciences Publications Ltd (2025). 10.7554/eLife.105213 PMC13537763 42684331

[42] Yu C.-H. et al., “Diesel2p mesoscope with dual independent scan engines for flexible capture of dynamics in distributed neural circuitry,” Nat. Commun. 12(1), 6639 (2021). NCAOBW 2041-1723 10.1038/s41467-021-26736-4 34789723 PMC8599518

[43] Sofroniew N. J. et al., “A large field of view two-photon mesoscope with subcellular resolution for in vivo imaging,” eLife 5, e14472 (2016). 10.7554/eLife.14472 27300105 PMC4951199

[44] Ota K. et al., “Fast, cell-resolution, contiguous-wide two-photon imaging to reveal functional network architectures across multi-modal cortical areas,” Neuron 109(11), 1810–1824.e9 (2021). NERNET 0896-6273 10.1016/j.neuron.2021.03.032 33878295

[45] Bounds H. A. et al., “All-optical recreation of naturalistic neural activity with a multifunctional transgenic reporter mouse,” Cell Rep. 42(8), 112909 (2023). 10.1016/j.celrep.2023.112909 37542722 PMC10755854

[46] Hira R. et al., “Reward-timing-dependent bidirectional modulation of cortical microcircuits during optical single-neuron operant conditioning,” Nat. Commun. 5(1), 5551 (2014). NCAOBW 2041-1723 10.1038/ncomms6551 25418042

[47] Carrillo-Reid L. et al., “Imprinting and recalling cortical ensembles,” Science 353(6300), 691–694 (2016). SCIEAS 0036-8075 10.1126/science.aaf7560 27516599 PMC5482530

[48] Yu P. et al., “Circuit-based understanding of fine spatial scale clustering of orientation tuning in mouse visual cortex,” bioRxiv (2025).

[49] Accanto N. et al., “Multiplexed temporally focused light shaping for high-resolution multi-cell targeting,” Optica 5(11), 1478–1491 (2018). 10.1364/OPTICA.5.001478

[50] Faini G. et al., “Ultrafast light targeting for high-throughput precise control of neuronal networks,” Nat. Commun. 14(1), 1888 (2023). NCAOBW 2041-1723 10.1038/s41467-023-37416-w 37019891 PMC10074378

[51] Inazawa K. et al., “Enhancement of optical sectioning capability of temporal focusing microscopy by using time-multiplexed multi-line focusing,” Appl. Phys. Express 14(8), 082008 (2021). APEPC4 1882-0778 10.35848/1882-0786/ac1387

[52] Ishikawa T. et al., “Adaptive optics with spatio-temporal lock-in detection for temporal focusing microscopy,” Opt. Express 29(18), 29021–29033 (2021). OPEXFF 1094-4087 10.1364/OE.432414 34615020

[53] Geiller T. et al., “Large-scale 3D two-photon imaging of molecularly identified CA1 interneuron dynamics in behaving mice,” Neuron 108(5), 968–983.e9 (2020). NERNET 0896-6273 10.1016/j.neuron.2020.09.013 33022227 PMC7736348

[r54] Griffiths V. A. et al., “Real-time 3D movement correction for two-photon imaging in behaving animals,” Nat. Methods 17(7), 741–748 (2020).1548-7091 10.1038/s41592-020-0851-7 32483335 PMC7370269

[r55] Judák L. et al., “Sharp-wave ripple doublets induce complex dendritic spikes in parvalbumin interneurons in vivo,” Nat. Commun. 13(1), 6715 (2022). NCAOBW 2041-1723 10.1038/s41467-022-34520-1 36344570 PMC9640570

[56] Villette V. et al., “Ultrafast two-photon imaging of a high-gain voltage indicator in awake behaving mice,” Cell 179(7), 1590–1608.e23 (2019). CELLB5 0092-8674 10.1016/j.cell.2019.11.004 31835034 PMC6941988

[57] Hossein Eybposh M. et al., “DeepCGH: 3D computer-generated holography using deep learning,” Opt. Express 28(18), 26636–26650 (2020). OPEXFF 1094-4087 10.1364/OE.399624 32906933

[58] Kishi K. E. et al., “Structural basis for channel conduction in the pump-like channelrhodopsin ChRmine,” Cell 185(4), 672–689.e23 (2022). CELLB5 0092-8674 10.1016/j.cell.2022.01.007 35114111 PMC7612760

[59] Ronzitti E. et al., “Submillisecond optogenetic control of neuronal firing with two-photon holographic photoactivation of chronos,” J. Neurosci. 37(44), 10679–10689 (2017). JNRSDS 0270-6474 10.1523/JNEUROSCI.1246-17.2017 28972125 PMC5666587

[60] Govorunova E. G. et al., “Natural light-gated anion channels: a family of microbial rhodopsins for advanced optogenetics,” Science 349(6248), 647–650 (2015). SCIEAS 0036-8075 10.1126/science.aaa7484 26113638 PMC4764398

[61] Govorunova E. G. et al., “Kalium channelrhodopsins are natural light-gated potassium channels that mediate optogenetic inhibition,” Nat. Neurosci. 25(7), 967–974 (2022). NANEFN 1097-6256 10.1038/s41593-022-01094-6 35726059 PMC9854242

[62] Vierock J. et al., “WiChR, a highly potassium-selective channelrhodopsin for low-light one- and two-photon inhibition of excitable cells,” Sci. Adv. 8(49), eadd7729 (2022). STAMCV 1468-6996 10.1126/sciadv.add7729 36383037 PMC9733931

[63] Accanto N. et al., “A flexible two-photon fiberscope for fast activity imaging and precise optogenetic photostimulation of neurons in freely moving mice,” Neuron 111(2), 176–189.e6 (2023). NERNET 0896-6273 10.1016/j.neuron.2022.10.030 36395773

[64] Forli A. et al., “Optogenetic strategies for high-efficiency all-optical interrogation using blue-light-sensitive opsins,” eLife 10, e63359 (2021). 10.7554/eLife.63359 34032211 PMC8177884

[65] Forli A. et al., “Two-photon bidirectional control and imaging of neuronal excitability with high spatial resolution in vivo,” Cell Rep. 22(11), 3087–3098 (2018). 10.1016/j.celrep.2018.02.063 29539433 PMC5863087

[66] Lim S. T. et al., “A novel targeting signal for proximal clustering of the Kv2.1 $K^{+}$ channel in hippocampal neurons,” Neuron 25(2), 385–397 (2000). NERNET 0896-6273 10.1016/S0896-6273(00)80902-2 10719893

[67] Histed M. H. Bonin V. Reid R. C., “Direct activation of sparse, distributed populations of cortical neurons by electrical microstimulation,” Neuron 63(4), 508–522 (2009). NERNET 0896-6273 10.1016/j.neuron.2009.07.016 19709632 PMC2874753

[68] Ersaro N. T. Yalcin C. Muller R., “The future of brain–machine interfaces is optical,” Nat. Electron. 6(2), 96–98 (2023). NEREBX 0305-2257 10.1038/s41928-023-00926-y

[69] Hira R., “Closed-loop experiments and brain machine interfaces with multiphoton microscopy,” Neurophotonics 11(3), 033405 (2024). 10.1117/1.NPh.11.3.033405 38375331 PMC10876015

[70] Tang X. et al., “Flexible brain–computer interfaces,” Nat. Electron. 6(2), 109–118 (2023). NEREBX 0305-2257 10.1038/s41928-022-00913-9

[r71] Saha S. et al., “Progress in brain computer interface: challenges and opportunities,” Front. Syst. Neurosci. 15, 578875 (2021). 10.3389/fnsys.2021.578875 33716680 PMC7947348

[r72] Gao X. et al., “Interface, interaction, and intelligence in generalized brain–computer interfaces,” Trends Cogn. Sci. 25(8), 671–684 (2021). TCSCFK 1364-6613 10.1016/j.tics.2021.04.003 34116918

[r73] Gallego J. A. Makin T. R. McDougle S. D., “Going beyond primary motor cortex to improve brain–computer interfaces,” Trends Neurosci. 45(3), 176–183 (2022). TNSCDR 0166-2236 10.1016/j.tins.2021.12.006 35078639

[r74] Rapeaux A. B. Constandinou T. G., “Implantable brain machine interfaces: first-in-human studies, technology challenges and trends,” Curr. Opin. Biotechnol. 72, 102–111 (2021). CUOBE3 0958-1669 10.1016/j.copbio.2021.10.001 34749248

[75] Andersen R. A. et al., “Exploring cognition with brain–machine interfaces,” Annu. Rev. Psychol. 73(1), 131–158 (2022). ARPSAC 0066-4308 10.1146/annurev-psych-030221-030214 34982594

[76] Mitani A. Dong M. Komiyama T., “Brain-computer interface with inhibitory neurons reveals subtype-specific strategies,” Curr. Biol. 28(1), 77–83.e4 (2018). CUBLE2 0960-9822 10.1016/j.cub.2017.11.035 29249656 PMC5760288

[77] Trautmann E. M. et al., “Dendritic calcium signals in rhesus macaque motor cortex drive an optical brain-computer interface,” Nat. Commun. 12(1), 3689 (2021). NCAOBW 2041-1723 10.1038/s41467-021-23884-5 34140486 PMC8211867

[78] Prsa M. Galiñanes G. L. Huber D., “Rapid integration of artificial sensory feedback during operant conditioning of motor cortex neurons,” Neuron 93(4), 929–939.e6 (2017). NERNET 0896-6273 10.1016/j.neuron.2017.01.023 28231470 PMC5330804

[79] Zhang Z. et al., “Closed-loop all-optical interrogation of neural circuits in vivo,” Nat. Methods 15(12), 1037–1040 (2018).1548-7091 10.1038/s41592-018-0183-z 30420686 PMC6513754

[80] Shang C.-F. et al., “Real-time analysis of large-scale neuronal imaging enables closed-loop investigation of neural dynamics,” Nat. Neurosci. 27(5), 1014–1018 (2024). NANEFN 1097-6256 10.1038/s41593-024-01595-6 38467902

[81] Muir J. Anguiano M. Kim C., “Neuromodulator and neuropeptide sensors and probes for precise circuit interrogation in vivo,” Science 385(6716), eadn6671 (2024). SCIEAS 0036-8075 10.1126/science.adn6671 39325905 PMC11488521

[82] Kleinlogel S. et al., “Emerging approaches for restoration of hearing and vision,” Physiol. Rev. 100(4), 1467–1525 (2020). PHREA7 0031-9333 10.1152/physrev.00035.2019 32191560

[r83] Ma R. et al., “The emerging field of oncolytic virus-based cancer immunotherapy,” Trends Cancer 9(2), 122–139 (2023). 10.1016/j.trecan.2022.10.003 36402738 PMC9877109

[84] Sahel J.-A. et al., “Partial recovery of visual function in a blind patient after optogenetic therapy,” Nat. Med. 27(7), 1223–1229 (2021).1078-8956 10.1038/s41591-021-01351-4 34031601

[85] Challis R. C. et al., “Adeno-associated virus toolkit to target diverse brain cells,” Annu. Rev. Neurosci. 45(1), 447–469 (2022). ARNSD5 0147-006X 10.1146/annurev-neuro-111020-100834 35440143

[86] Chan K. Y. et al., “Engineered AAVs for efficient noninvasive gene delivery to the central and peripheral nervous systems,” Nat. Neurosci. 20(8), 1172–1179 (2017). NANEFN 1097-6256 10.1038/nn.4593 28671695 PMC5529245

[87] Ramezani M. et al., “High-density transparent graphene arrays for predicting cellular calcium activity at depth from surface potential recordings,” Nat. Nanotechnol. 19(4), 504–513 (2024). NNAABX 1748-3387 10.1038/s41565-023-01576-z 38212523 PMC11742260

[88] Cabrera L. Y. Weber D. J., “Rethinking the ethical priorities for brain–computer interfaces,” Nat. Electron. 6(2), 99–101 (2023). NEREBX 0305-2257 10.1038/s41928-023-00928-w

[89] Tsutsumi S. Hayashi-Takagi A., “Optical interrogation of multi-scale neuronal plasticity underlying behavioral learning,” Curr. Opin. Neurobiol. 67, 8–15 (2021). COPUEN 0959-4388 10.1016/j.conb.2020.07.002 32768886

[90] Pachitariu M. et al., “Suite2p: beyond 10,000 neurons with standard two-photon microscopy,” bioRxiv (2016).

[91] Giovannucci A. et al., “CaImAn an open source tool for scalable calcium imaging data analysis,” eLife 8, e38173 (2019). 10.7554/eLife.38173 30652683 PMC6342523

[92] Pnevmatikakis E. A. Giovannucci A., “NoRMCorre: an online algorithm for piecewise rigid motion correction of calcium imaging data,” J. Neurosci. Methods 291, 83–94 (2017). JNMEDT 0165-0270 10.1016/j.jneumeth.2017.07.031 28782629

[93] Pnevmatikakis E. A. et al., “Simultaneous denoising, deconvolution, and demixing of calcium imaging data,” Neuron 89(2), 285–299 (2016). NERNET 0896-6273 10.1016/j.neuron.2015.11.037 26774160 PMC4881387

[94] Bowen Z. et al., “NeuroART: real-time analysis and targeting of neuronal population activity during calcium imaging for informed closed-loop experiments,” eNeuro 11(10), ENEURO.0079-24.2024 (2024). 10.1523/ENEURO.0079-24.2024 PMC11485737 39266327

[95] Sheng W. et al., “Real-time image processing toolbox for all-optical closed-loop control of neuronal activities,” Front. Cell. Neurosci. 16, 917713 (2022). 10.3389/fncel.2022.917713 35865111 PMC9294372

[96] Zhu F. et al., “A deep learning framework for inference of single-trial neural population dynamics from calcium imaging with subframe temporal resolution,” Nat. Neurosci. 25(12), 1724–1734 (2022). NANEFN 1097-6256 10.1038/s41593-022-01189-0 36424431 PMC9825112

[97] Lee W.-H. et al., “Identifying distinct neural features between the initial and corrective phases of precise reaching using AutoLFADS,” J. Neurosci. 44(20), e1224232024 (2024). JNRSDS 0270-6474 10.1523/JNEUROSCI.1224-23.2024 38538142 PMC11097258

[98] Pandarinath C. et al., “Inferring single-trial neural population dynamics using sequential auto-encoders,” Nat. Methods 15(10), 805–815 (2018).1548-7091 10.1038/s41592-018-0109-9 30224673 PMC6380887

[99] Prince L. Y. et al., “Parallel inference of hierarchical latent dynamics in two-photon calcium imaging of neuronal populations,” bioRxiv (2021).

[100] Pellegrino A. Stein H. Cayco-Gajic N. A., “Dimensionality reduction beyond neural subspaces with slice tensor component analysis,” Nat. Neurosci. 27(6), 1199–1210 (2024). NANEFN 1097-6256 10.1038/s41593-024-01626-2 38710876 PMC11537991

[101] Williams A. H. et al., “Unsupervised discovery of demixed, low-dimensional neural dynamics across multiple timescales through tensor component analysis,” Neuron 98(6), 1099–1115.e8 (2018). NERNET 0896-6273 10.1016/j.neuron.2018.05.015 29887338 PMC6907734

[102] Mathis A. et al., “DeepLabCut: markerless pose estimation of user-defined body parts with deep learning,” Nat. Neurosci. 21(9), 1281–1289 (2018). NANEFN 1097-6256 10.1038/s41593-018-0209-y 30127430

[103] Kane G. A. et al., “Real-time, low-latency closed-loop feedback using markerless posture tracking,” eLife 9, e61909 (2020). 10.7554/eLife.61909 33289631 PMC7781595

